# Regression of Atherosclerosis in ApoE−/− Mice Via Modulation of Monocyte Recruitment and Phenotype, Induced by Weekly Dosing of a Novel “Cytotopic” Anti‐Thrombin Without Prolonged Anticoagulation

**DOI:** 10.1161/JAHA.119.014811

**Published:** 2020-07-02

**Authors:** Daxin Chen, Ke Li, Sam Festenstein, Julieta Karegli, Hannah Wilkinson, Hugh Leonard, Lin‐Lin Wei, Ning Ma, Min Xia, Henry Tam, Jian‐an Wang, Qingbo Xu, John H. McVey, Richard A. G. Smith, Anthony Dorling

**Affiliations:** ^1^ Department of Inflammation Biology School of Immunology and Microbial Sciences King’s College London, Guy’s Hospital London United Kingdom; ^2^ Core Research Laboratory the Second Affiliated Hospital, School of Medicine Jiaotong University Xi’an China; ^3^ Thrombosis Research Institute London United Kingdom; ^4^ Department of Imaging Imperial College Healthcare NHS Trust Charing Cross Hospital London United Kingdom; ^5^ Department of Cardiology Second Affiliated Hospital of Zhejiang University School of Medicine Hangzhou China; ^6^ Cardiovascular Division King’s College London James Black Centre London United Kingdom; ^7^ School of Bioscience & Medicine Faculty of Health and Medical Sciences University of Surrey Guildford United Kingdom

**Keywords:** atherosclerosis, regression, thrombin, thrombin inhibitor, Atherosclerosis, Animal Models of Human Disease, Inflammation, Translational Studies

## Abstract

**Background:**

Anticoagulants induce atherosclerosis regression in animal models but exploiting this clinically is limited by bleeding events. Here we test a novel thrombin inhibitor, PTL060, comprising hirulog covalently linked to a synthetic myristoyl electrostatic switch to tether to cell membranes.

**Methods and Results:**

ApoE−/− mice were fed chow or high‐fat diets, before transplantation of congenic aortic segments or injection of PTL060, parental hirulog, control saline, or labeled CD11b positive cells. Aortic transplants from transgenic mice expressing anticoagulants on endothelium did not develop atherosclerosis. A single intravenous injection of PTL060, but not hirulog inhibited atheroma development by >50% compared with controls when assessed 4 weeks later. Mice had prolonged bleeding times for only one seventh of the time that PTL060 was biologically active. Repeated weekly injections of PTL060 but not hirulog caused regression of atheroma. We dissected 2 contributory mechanisms. First, the majority of CCR2+ (C‐C chemokine receptor type 2+) monocytes recruited into plaques expressed CCR7 (C‐C chemokine receptor type 7), ABCA1 (ATP‐binding cassette transporter – 1), and interleukin‐10 in PTL060 mice, a phenotype seen in <20% of CCR2+ recruits in controls. Second, after several doses, there was a significant reduction in monocyte recruits, the majority of which were CCR2‐negative with a similar regression‐associated phenotype. Regression equivalent to that induced by intravenous PTL060 was induced by adoptive transfer of CD11b+ cells pre‐coated with PTL060.

**Conclusions:**

Covalent linkage of a myristoyl electrostatic switch onto hirulog in PTL060 uncouples the pharmacodynamic effects on hemostasis and atherosclerosis, such that plaque regression, mediated predominantly via effects on monocytes, is accompanied by only transient anticoagulation.

**Nonstandard Abbreviations and Acronyms**
ABCA1: ATP‐binding cassette transporter – 1
BL/6: C57BL/6 mice
CCL2: chemokine (C‐C motif) ligand 2
CCR2: C‐C chemokine receptor type 2
CCR7: C‐C chemokine receptor type 7
CD31‐Hir‐Tg: a strain of transgenic mice expressing membrane tethered hirudin under CD31 promoter
EC: endothelial cell
HFD: high‐fat diet
HLL: chemically modified hirulog analogue
hTFPI: human tissue factor pathway inhibitor
iNOS: inducible NO synthase
MIF: macrophage migration inhibitory factor
PAR: protease‐activated receptor
SMC: smooth muscle cell
TF: tissue factor

**Clinical Perspective**

**What Is New?**

- We have developed a novel direct anti‐thrombin inhibitor specifically to target inflammatory processes, by covalently linking a synthetic myristoyl electrostatic switch (for cell‐membrane localization) to hirulog.
- Upon intravenous injection, it has the same anti‐coagulant profile as equimolar hirulog, but the membrane‐localizing component promotes prolonged localization on circulating leukocytes and vascular endothelium.
- This novel therapeutic induces regression of atherosclerosis in ApoE−/− mice after weekly intravenous dosing, by mechanisms that are dependent on this pattern of prolonged binding to cells within the vasculature; throughout treatment, mice are systemically anticoagulated for only 1 day out of 7.

**What Are the Clinical Implications?**

- For the first time we have defined a way to uncouple the effects of hirulog on hemostasis from its effects on atheroma formation.
- Alongside our descriptions of the mechanisms through which atheroma regression is induced, our findings should provide a foundation for the development of strategies to safely harness the powerful anti‐inflammatory effects of therapeutics that inhibit coagulation proteases, without adverse events related to bleeding.

Atherosclerosis is a chronic inflammatory disease that causes coronary artery, peripheral vascular and cerebrovascular disease. It is a major cause of death in the Western world. Important early steps in atherogenesis, in the context of a high lipid microenvironment include secretion of chemokines such as CCL2 (chemokine [C‐C motif] ligand 2) and MIF (macrophage migration inhibitory factor),^1^ by activated endothelial cells (ECs) and smooth muscle cells (SMCs).^2^, ^3^ These promote infiltration of monocytes into the subendothelial space, where they become macrophages and take up very‐low‐density lipoprotein and low‐density lipoprotein to become foam cells, initiating the process of atheroma formation.

Coagulation proteases, such as thrombin, signal though PAR (protease‐activated receptors) as well as catalyzing fibrin formation and are known to play a role in this process. Increased activity of TF (tissue factor), the 47‐Kd cell membrane‐bound glycoprotein that initiates the serine protease cascade, is seen in the neointima and underlying media of atherosclerotic plaques^4^, ^5^, ^6^ and TF is expressed by EC,^7^ monocytes/macrophages^8^ and SMC.^9^

In previous work, we crossed a strain of transgenic mice expressing a membrane‐tethered hTFPI (human tissue factor pathway inhibitor) fusion protein on α‐smooth muscle actin (SMA)^+^ cells (called [α‐TFPI‐Tg] mice)^10^ with ApoE^−/−^ mice to generate a new strain (called ApX4). These mice were resistant to atheroma formation.^11^ In dissecting the mechanism of resistance, we showed that TF expression by SMC was necessary to generate MIF, via generation of thrombin and signaling primarily through PAR‐1. Inhibition of either the TF, thrombin‐mediated PAR‐1 signaling or MIF secretion prevented atherosclerosis in mice fed either a high‐fat diet (HFD) or a regular chow‐based diet.

One of the observations from that study was that MIF continued to be secreted by EC in ApX4 mice fed an HFD, beneath which small atherosclerotic plaques developed.^11^ This suggested that targeting SMC with hTFPI was not completely efficient at inhibiting atheroma. In this new study, we explored how transgenically‐expressed tethered anticoagulants on EC impacted on atherosclerosis development, and assessed the translational potential of a novel thrombin inhibitor containing the potent peptide hirulog (a direct thrombin inhibitor), that has been chemically modified into a hirulog analogue (HLL) to accept a lipid membrane‐binding anchor or “cytotopic” tail. This new compound is called PTL060 (thrombalexin‐3). PTL060 has previously been localized within organs before transplantation to successfully inhibit thrombosis and rejection in several models.^12^, ^13^, ^14^ In the process we describe an unpredicted impact of PTL060 on the phenotype of monocytes recruited into atherosclerotic plaques, by 2 interrelated pathways, one of which occurs by virtue of its ability to tether directly to monocytes. We also provide a mechanistic insight into the role that thrombin appears to play in driving plaque progression, as evidenced by the regression seen when PTL060 is administered systemically.

## Methods

The data that support the findings of this study are available from the corresponding author upon reasonable request.

### Mice and In Vivo Procedures

C57BL/6J (BL/6) mice were purchased from Harlan UK Ltd (Bicester, UK) and ApoE−/− mice were from the Jackson Laboratory (Bar Harbor, Maine 04609, USA). CD31‐TFPI ‐Tg and a second strain of transgenic mice expressing membrane tethered hirudin under CD31 promoter (CD31‐Hir‐Tg)^15^ mice were bred in house. Mice were housed in a temperature‐controlled Specific Pathogen‐Free environment at 22 to 24°C and all animal surgical protocols, animal experiments and care were approved by the local ethics committee and the UK Home Office.

To assess the distribution of PTL060, male BL/6 mice weighing 25 to 28 g (n=6 per group) were injected intravenously through a tail vein with either PTL060 (10 μg/g in 100 μL saline), equimolar HLL (5 μg/g) or saline. At 5, 30 minutes and 2, 4, 6, 24, and 48 hours mice were euthanized to collect citrated whole blood for separation into cells and plasma and to harvest aortas for immunofluorescence analysis.

Bleeding times were assessed as previously described.^15^ Briefly, mice were anesthetized and placed in a restrainer (Becton Dickinson), before a distal 3‐mm segment of tail was severed with a razor blade. The tail was immediately immersed in 0.9% saline at 37°C. Bleeding time was defined as the time required for bleeding to stop. Experiments were terminated at this time or at 20 minutes.

For all atherosclerosis work, male ApoE−/− mice, from the age of 6 weeks, were fed with an HFD consisting 35 kcal% fat, 1.25% cholesterol, and 0.5% cholic acid (Special Diet Services, Essex, UK). Aortas were transplanted 2 weeks after starting the HFD, using a sleeve anastomosis technique described previously.^11^ Briefly, 5 mm of the segment of the infrarenal donor aorta, flushed with 300 μL of saline containing 50 U of heparin, was transplanted into ApoE−/− recipient abdomen aortas (N=6 per group). Blood flow was confirmed by direct inspection after removal of the clamps. Mice were fed an HFD for 6 to 12 weeks post‐transplantation before the experiment was terminated (Figure S1A). To assess prevention of atheroma, mice (n=6 per group) received a single injection of PTL060 or controls by tail vein, and the experiments terminated 1 to 3 weeks later (Figure S1B). For regression experiments, baseline groups (n=6) were fed an HFD to the age of 22 weeks before the mice were euthanized. Experimental or control groups (n=3–6 per group) received weekly injections by tail vein for 3 to 6 weeks, beginning at age of 22 weeks before the experiments were terminated when mice were aged 25 to 28 weeks (Figure S1C).

### Cell Isolation and Labeling

Leukocytes were isolated from the blood of mice aged 8 to 10 weeks, using anti‐CD11b MicroBeads (Miltenyi Biotec Ltd, Surrey, UK) according to manufacturers’ instructions. For cell labeling, 2×10^7^ CD11b+ cells were incubated with 4×10^−6^ mol/L of either PKH26 (red) or PKH2 (green) fluorescent dyes (Sigma, UK) for 5 minutes at 25°C according to manufacturer's protocols, with the reaction stopped using 1% BSA in PBS followed by 3 washes. Each recipient mouse received 0.5×10^6^ cells by intravenous injection; in some experiments, the cells were incubated with PTL060 (100 μmol/L in 0.5 mL) or equimolar controls for 30 minutes at room temperature and washed 3 times before immediate injection. Cell viability was confirmed immediately before transfer by trypan blue extraction.

For specific viability assays, murine bone marrow cells were incubated for 5 days in 6‐well plates, counted, and re‐seeded at 2×10^5^ cells/mL in 24 well plates with 25 ng/mL macrophage colony stimulating factor. After 1 day, media was replaced with new DMEM/FCS containing different concentrations of PTL060 (or a fixed volume of control PBS), and incubated for 30 to 120 minutes, before assessment by flow cytometry (see below).

### Histological Analysis

Atherosclerotic lesions were evaluated as previously described.^11^ Simply, the entire length of the aorta was perfused with PBS, dissected using a dissecting microscope, longitudinally opened and stained with Oil Red O solution (Sigma, UK) for 30 minutes, before being photographed with a digital camera (DSC‐W320, Sony, Japan). The total aortic area and lesional area were measured by using Image J. Aortas from every animal were assessed. To assess lesions in the aortic sinus, hearts were embedded in paraffin, sectioned through the aortic root and incubated with elastin/van Gieson stain using the AccustainTM Elastin Stain kit (Sigma). Sections were examined on an Olympus U‐ULH optical microscope (Olympus Optical Co. Ltd, Tokyo, Japan). Atheromatous lesional and total aortic root area was determined using Image‐Pro Plus TM software version 4.0 (Media Cybernetics, Silver Spring, USA). At least 3 random sections were examined from each mouse in all groups.

Immunohistology of frozen cross sections were prepared and examined as previously described.^11^ Briefly, isolated tissues were snap‐frozen and embedded in OCT compound (VWR International, Dorset, UK), sectioned at 5‐μm thickness and fixed in methanol at −20°C. Frozen sections were immersed in 1% bovine serum albumin–phosphate‐buffered saline (BSA‐PBS) for 30 minutes and then incubated overnight at 4°C with one or more of the following antibodies: rabbit polyclonal antibody to CD68, iNOS (inducible NO synthase), CD206, TNFα (tumor necrosis factor alpha) MIF, C‐C chemokine receptor type (CCR)7, ABCA1 (ATP‐binding cassette transporter – 1) (all from Abcam, Cambridge, UK), or hirudin (Biobyt, Cambridge, UK) or CCL2 (Lifespan BioScience, Inc., WA 98121, USA); goat polyclonal antibody to CD31 (Santa Cruz Biotechnology, Texas 75220, USA); rat anti‐mouse CD68, CD11b (Serotec, Oxford, United Kingdom), CD31, interferon gamma (IFNγ) (BD Bioscience Pharmingen, Oxford, United Kingdom), Ly‐6G (BioLegend, London, UK), interleukin‐10 (Abcam) or biotinylated anti‐HLL^14^; mouse anti‐CCR2 (C‐C chemokine receptor type 2) (Abcam). The following were used as isotype controls; goat anti‐rat antibodies to IgG2a, IgG2b (BD Bioscience, Berkshire, UK) and polyclonal rabbit IgG (Abcam). The following anti‐IgG FITC or TRITC‐conjugated antibodies were used: sheep anti‐mouse, rabbit anti‐rat, goat anti‐rabbit and rabbit anti‐goat (all from Sigma). Fluorescein‐conjugated streptavidin (Jackson Immunoresearch, Cambridge, UK) was used to detect the anti‐HLL. Stained sections were mounted in Vectashield with DAPI (Vector Laboratories Inc, CA USA). Sections were directly captured and examined by a Leica DMIRBE confocal microscope (Leica, Wetzlar, Germany) equipped with Leica digital camera AG and a confocal laser scanning system with excitation lines at 405, 488, 543, and 560 nm at magnifications 10×/0.40CS and 20×/0.70IMM (Leica, Planapo, Wetzlar, Germany). Images were processed using Leica‐TCS‐NT software associated with the Leica confocal microscope. All immunohistochemistry was performed at 22°C. Quantification of staining was achieved by expressing the area of positive staining as a ratio of the total lesion area, calculated using Image‐Pro Plus TM, version 4.0. All quantification was performed by members of the team blinded to the identity of the sections. For estimations of positive stained area, average measurements were derived from examination of at least six random sections from each tissue sample.

To detect macrophage‐derived foam cells, frozen sections of aortic sinus were analyzed by a combination of Oil Red O staining and CD68 immunostaining. Sections were incubated with rat anti‐mouse CD68 antibody (overnight at 4°C) and goat anti‐rat antibody (1 hour at room temperature) before staining with filtered Oil Red O solution (0.5% in propylene glycol, Sigma) for 15 minutes at room temperature.

### Plasma Assays

Anticoagulated whole blood (EDTA 30 mmol/L pH8) was separated into plasma and cells by centrifugation (14 000*g* for 10 minutes). Plasma TNFα, IFN‐γ, MIF, and CCL2 were detected using separate specific ELISA kits (R&D Systems, Abingdon, UK) according to the manufacturers’ instructions. Total cholesterol, high‐density lipoprotein and low‐density lipoprotein were determined using kits from Cell Biolabs, and triglycerides with a kit from Abcam, (both Cambridge, UK) according to the manufacturer's protocol. Data were derived from triplicate analysis of each sample.

Thrombin clotting times were measured in 3.2% trisodium citrated plasma according to the protocol of Ignjatovic.^16^ Briefly, 100 μL mouse plasma was incubated with 2.5 U of human thrombin in a total volume of 300 μL (Enzyme Research Laboratories, Swansea, UK) at 37°C, and the time for a clot to form was measured (n=6 per group). For some experiments plasma was further centrifuged (20 000*g* for 10 minutes) to minimize the presence of extracellular vesicles.

### Flow Cytometry

The cells obtained from whole blood were washed twice in PBS with 2% FCS before staining with either anti‐CD11b‐FITC (Abcam) or anti‐CD41‐FITC (eBioscience) with biotinylated anti‐HLL followed by Streptavidin‐PE (Bio‐Rad). Cells were then washed twice before analysis on a BD FACSCALIBUR with CellQuest Pro software. Erythrocytes were identified by forward/side scatter profile.

For viability assays, cells were washed twice with PBS and then incubated with Fixable LIVE/DEAD Near‐IR fluorescent reactive dye (Thermo Fisher Scientific, Paisley, Renfrewshire, UK) for 30 minutes at 4°C. Cells were washed, fixed for 15 minutes in 1% paraformaldehyde, then washed with PBS‐5% FCS and stored at 4°C before acquisition and analysis within 24 hours on an LSRII/Fortessa flow cytometer at the BRC Flow Cytometry Laboratory, King's College London with FlowJo software (Treestar Inc). Macrophages identified by forward/side scatter profile.

### SMC‐MIF/CCL2 Release Assay In Vitro

SMCs, cultured as previously described^11^ and seeded at a density of 1×10^6^ cells/well of a 24‐well plate were serum‐starved for 24 hours before addition of PTL060 (100 μmol/L) for 1 hour, followed by PAR agonists or antagonists (all from Enzyme Research Laboratories) for 12 hours, followed by thrombin 10 nmol/L or active site inhibited thrombin (Enzyme Research Laboratories) for 48 hours, before collection of supernatants. Chemokines were measured by ELISA according to the manufacturer's instructions (R&D Systems, Abingdon, UK). Data were derived from triplicate analysis of each sample.

### Statistical Analysis

Statistical analysis was performed with GraphPad Prism 8 software. Comparison of a single factor between 2 groups is by unpaired Student *t‐*test. One‐ or two‐way ANOVA was used as appropriate when making comparisons of ≥2 factors or between multiple groups. Data are presented either as mean±SEM or as box plots with median and interquartile range. The value of *P* that was considered significant was adjusted for multiple comparisons and listed in figure legends.

## Results

### Anticoagulants Transgenically Localized to EC Completely Inhibit Vessel Wall Expression of Chemokines and Prevent Formation of Atheroma

**Figure 1:**
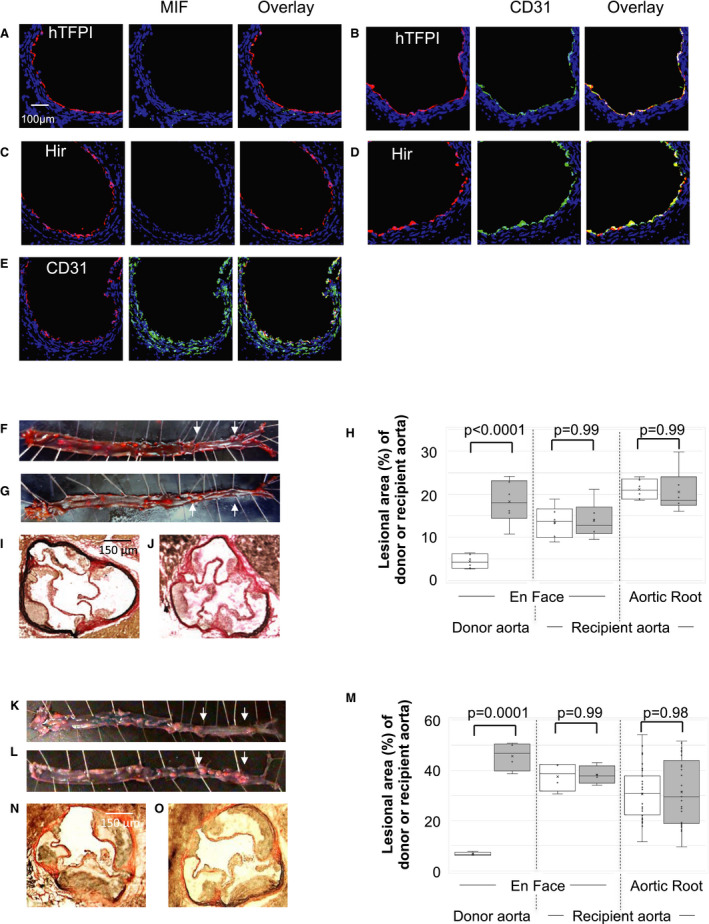
Inhibition of TF or thrombin on endothelial cells abolishes macrophage migration inhibitory factor expression in vascular wall and prevents formation of atheroma. **A** through **E**, Three color immunofluorescence images of sections through donor aortas, 6 to 12 weeks post‐transplantation. Recipients were ApoE−/− mice, fed a high‐fat diet for 2 weeks from age 6 weeks, before transplantation of aorta from CD31‐TFPI‐Tg (**A** and **B**), CD31‐Hir‐Tg (**C** and **D**) or C57BL/6 mice (**E**). Blue—nuclear stain DAPI. Red—anti‐human tissue factor pathway inhibitor (**hTFPI‐ A** and **B**), anti‐hirudin (Hir‐**C** and **D**) or anti‐CD31 (**E**). Green—macrophage migration inhibitory factor (MIF‐ **A**,** C**, and **E**) or CD31 (**B** and **D**). Each panel of 3 images shows consecutive sections. **F** through **J**, Analysis of atheroma development in whole aorta (**F**,** G**, and **H**) and aortic root (**H**,** I**, and **J**) after a high‐fat diet for 6 weeks post‐transplantation. **F** and **G**, Representative Oil Red O‐stained en face preparations of aorta from ApoE−/− mice transplanted with aorta from CD31‐TFPI‐Tg (**F**) or BL/6 (**G**) mice. The transplanted section is highlighted by arrows. **H**, Quantitative assessments show the area occupied by atheroma, assessed at 3 different sites (as indicated) as a proportion of the total area (n=6 males each group) in ApoE−/− mice transplanted with aortas from CD31‐TFPI‐Tg (white bars) or BL/6 (grey bars) donors. Graphs show box plots with median with interquartile range with whiskers showing upper and lower limits and outliers indicated as single data points. Means are represented with “x”. **I** and **J**, Representative light photomicrographs of elastic/van Gieson stained sections from aortic root of mice transplanted with aortas from CD31‐TFPI‐Tg (**I**) or BL/6 (**J**) mice. **K** through **O**, Analysis of atheroma development in the whole aorta (**K**,** L**, and **M**) and aortic root (**M**,** N**, and **O**) after a high‐fat diet for 12 weeks post‐transplantation. **K** and **L**, representative Oil Red O‐stained en face preparations of aorta from ApoE−/− mice transplanted with aorta from CD31‐Hir‐Tg (**K**) or BL/6 (**L**) mice. The transplanted section is highlighted by arrows. **M**, Quantitative assessments show the area occupied by atheroma, assessed at 3 different sites (as indicated) as a proportion of the total area (n=6 males each group) in ApoE−/− mice transplanted with aortas from CD31‐Hir‐Tg (white bars) or BL/6 (grey bars) donors. Graphs show box plots with median with interquartile range with whiskers showing upper and lower limits and outliers indicated as single data points. Means are represented with “x”. **N** and **O**, Representative light photomicrographs of elastic/van Gieson stained sections from aortic root of mice transplanted with aortas from CD31‐Hir‐Tg (**N**) or BL/6 (**O**) mice. Quantitative analyses were performed by a member of the team blinded to the mouse strain. Comparisons of plaque development analyzed by repeated measures 2‐way ANOVA. Because multiple comparisons were made from these animals, *P*<0.02 is statistically significant. hFTPI indicates human tissue factor pathway inhibitor; MIF, macrophage migration inhibitory factor; and TF, tissue factor.

To assess the impact of expressing human TFPI fusion protein on EC alone, we used the congenic aortic transplant model previously described,^11^ and compared the extent of atheroma development in transplanted aortas from CD31‐TFPI‐Tg mice (expressing hTFPI transgene on EC^15^) and BL/6 mice. The recipients were 8‐week‐old ApoE−/− mice fed an HFD for 2 weeks before the transplant, and the experiment was terminated 6 weeks after the transplant (Figure S1). In the aortic transplants from CD31‐TFPI‐Tg mice, MIF expression was absent through the entire wall of the transplanted vessel, not just the EC (Figure 1A and 1B), and atheroma formation was significantly attenuated in the transplanted donor segment (Figure 1F and 1H). In contrast and as previously reported, control BL/6 aortic transplants developed exaggerated lesions, associated with MIF expression in all layers of the vascular wall (Figure 1E, 1G, 1H, and 1J). The atherosclerosis that developed in the recipient aortas was independent of the type of donor aorta transplanted (Figure 1G, 1H, and 1I).

Next we transplanted aortas from a second transgenic strain (CD31‐Hir‐Tg),^15^ expressing a tethered hirudin fusion protein on EC (Figure S1), and these showed similar suppression of MIF expression throughout the vessel wall and were similarly resistant to atheroma development (Figure 1C, 1D, 1K through 1O), indicating that inhibition of thrombin and TF on EC was functionally equivalent, entirely consistent with our previously published results.^11^ In addition to MIF, CCL‐2 expression was completely suppressed throughout the vessel walls of transplants from both transgenic strains (Figure S2). These data indicate that inhibiting thrombin generation (by TFPI) or the enzymatic activity of thrombin (by hirudin) on transgenic EC after transplantation into ApoE−/− mice completely suppresses MIF and CCL‐2 expression throughout the entire vascular wall and prevents atheroma formation.

### Intravenous Injection of PTL060, a Novel Tethered Therapeutic Anti‐Thrombin

**Figure 2:**
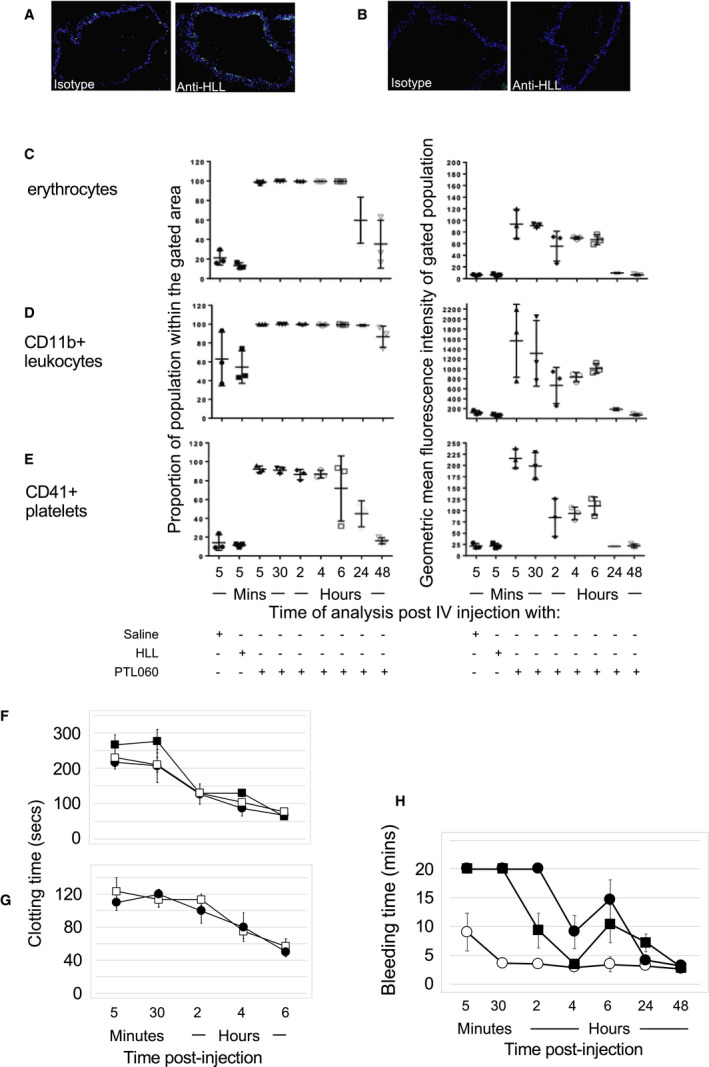
Impact of intravenous PTL060. **A** and **B**, Two color immunofluorescence images of cross sections through aorta harvested at 6 hours post‐intravenous injection of 10 μg/g PTL060 (**A**) or equimolar (5 μg/g) chemically modified hirulog analogue HLL (**B**) stained with isotype control or anti‐HLL antibody as indicated. Blue ‐DAPI. (Please note Sections examined at all other time points showed less evidence of binding by PTL060). **C** through **E**, Flow cytometric assessment of binding to erythrocytes (**C**), CD11b+ leukocytes (**D**) and platelets (gated on CD41+) (**E**) obtained from mice given either saline control, chemically modified hirulog analogue HLL (2.5 μg/g), or PTL060 (5 μg/g). Graphs show percentage of population binding anti‐HLL antibody (left column) and the geometric mean of the fluorescence intensity of binding (right column). Samples were taken from mice at the time points post‐injection as indicated. n=3 per group. **F** and **G**, Thrombin clotting times (seconds±SEM) in plasma. Blood was collected into citrated tubes at the times specified under terminal anesthesia before spinning at 15 000*g* for 10 minutes to separate out cellular components and plasma. Thrombin times performed by adding 25 U (**F**) or 50 U (**G**) thrombin to 100 μL of plasma and recording time for a fibrin clot to form. Mice (n=3 per group) injected with PTL060 (5 μg/g—filled squares) or equimolar dose of chemically modified hirulog analogue HLL (2.5 μg/g—circles). Plasma from mice treated with PTL060 was centrifuged for a further 20 minutes at 10 000*g*, to remove any membrane bound PTL060, before repeating assessments (open squares). **H**, Graph depicting tail bleeding times in minutes±SEM at various times after intravenous injection of control phosphate buffered saline (open circles), PTL060 10 μg/g (squares) or equimolar (5 μg/g) chemically modified hirulog analogue HLL (closed circles). N=6 per group. Mouse euthanized at 20 minutes if tail still bleeding.

After intravenous administration of PTL060 into BL/6 mice, linear deposition of the anticoagulant moiety could be found on the luminal surface of the aorta mice several hours later (Figure 2A). This pattern of staining was never seen after injection of the parental (untailed) chemically modified hirulog analogue HLL (Figure 2B). PTL060 also quickly attached to the lipid membranes of circulating erythrocytes, leukocytes, and platelets (Figure 2C through 2E), maintaining stable levels of binding between 2 and 6 hours post‐injection, before reducing between 24 and 48 hours post‐injection. No binding was ever seen after injection of HLL. In vitro viability assays, performed by incubating bone marrow‐derived macrophages with increasing concentrations of PTL060 for 30 to 120 minutes confirmed no evidence of toxicity (Table S1).

Thrombin clotting times of citrated plasma were prolonged for >6 hours post injection of PTL060, indicating the presence of a thrombin inhibitor, and these were not statistically different to thrombin clotting times after injection of an equimolar amount of HLL (Figure 2F and 2G). This was associated with prolonged tail bleeding times (Figure 2H) lasting for ≈24 hours, with bleeding times in PTL060‐treated mice not statistically different from those recorded in mice given an equimolar concentration of HLL (Figure 2H). Thus, intravenous injection of PTL060 resulted in rapid uptake onto the membranes of circulating cells and platelets, with detectable deposition on EC a few hours later. Despite this, the thrombin inhibitory activity detected in plasma was indistinguishable from that seen after injection of the HLL, and mice showed prolonged bleeding for 24 hours.

**Figure 3:**
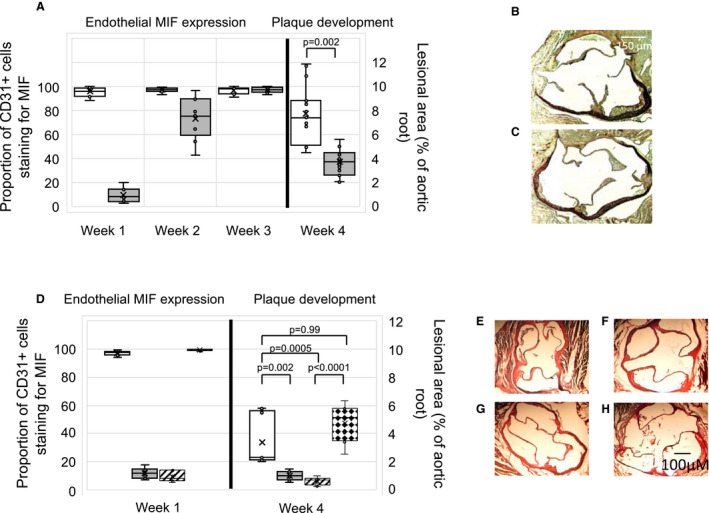
Intravenous PTL060 inhibits MIF (macrophage migration inhibitory factor) and prevents atherosclerosis. **A**, Quantitative impact of PTL060 on MIF expression by endothelium (left axis), represented as the proportion of CD31+ cells staining for MIF, plotted against time or development of atheroma (right axis) 4 weeks post‐injection. Mice (n=6) given either PBS control (white) or PTL060 10 μg/g (grey) by IV injection, 2 weeks after starting a high‐fat diet and analyzed at the time points indicated. Graphs show box plots with median with interquartile range with whiskers showing upper and lower limits and outliers indicated as single data points. Means are represented with “x”. Comparisons of plaque development analyzed by unpaired t test. *P*<0.05 is statistically significant. **B** and **C**, Representative light photomicrographs of elastic/van Gieson stained sections from aortic root of ApoE−/− mice treated with PBS (**B**) or 10 μg/g PTL060 (**C**). **D**, Quantitative impact of PTL060 on MIF expression 1‐week post injection (left axis), represented as the proportion of CD31+ cells staining for MIF, or development of atheroma (right axis) 4‐weeks post‐injection. Mice (n=6) given either PTL060 2.5 μg/g (white bars), PTL060 5 μg/g (grey bars), PTL060 10 μg/g (striped bars), or chemically modified hirulog analogue HLL 5 μg/g (diamond bars) by intravenous injection, 2 weeks after starting a high‐fat diet and analyzed at the time points indicated. Chemically modified hirulog analogue HLL 5 μg/g is equimolar to PTL060 10 μg/g. Graphs show box plots with median with interquartile range with whiskers showing upper and lower limits and outliers indicated as single data points. Means are represented with “x”. Comparisons of plaque development analyzed by repeated measures one‐way ANOVA. *P*<0.05 is statistically significant. **E** through **H**, Representative light photomicrographs of elastic/van Gieson stained sections from aortic root of ApoE−/− mice treated with PTL060 2.5 μg/g (**E**), 5 μg/g (**F**), 10 μg/g (**G**), or chemically modified hirulog analogue HLL 5 μg/g (**H**). MIF indicates macrophage migration inhibitory factor.

We assessed the differential impact of PTL060 and HLL on expression of MIF by the vasculature, as a biomarker of potential efficacy at suppressing atheroma formation. A single intravenous injection of PTL060 was accompanied by complete suppression of MIF expression throughout the vessel wall for almost 1‐week (Figure S2D; Figure 3A), which was dose‐dependent (Figure 3D). This effect was never seen in controls given saline (Figure S2E) or in mice administered an equimolar dose of HLL (Figure 3D). This prolonged biological effect of PTL060 is consistent with our previous demonstration that, once bound to endothelium, it remains detectable for >4 days.^14^ In vitro experiments confirmed that thrombin‐mediated chemokine expression was primarily via PAR‐1 and that PTL060 completely inhibited this (Figure S3).

These data indicate that equimolar doses of PTL060 and HLL induce similar degrees of systemic thrombin inhibition lasting ≈24 hours, but only PTL060 promotes prolonged suppression of MIF expression by vessel wall cells.

### Impact of PTL060 on Atherosclerosis

A single injection of PTL060 caused significant inhibition of atheroma formation in ApoE−/− mice fed an HFD for 2 weeks before, and 4 weeks after the injection (Figure 3A, 3B, and 3C). This effect was dose‐dependent (Figure 3D and 3E through 3H), and only occurred with doses that inhibited MIF expression for up to 1 week (Figure 3A and 3D; Figure S2D). It was not seen in mice administered equimolar doses of HLL (Figure 3D and 3H). Thus, a membrane tethered thrombin inhibitor can replicate the impact of a transgenically expressed membrane tethered thrombin inhibitor by suppressing the development of atherosclerosis.

**Figure 4:**
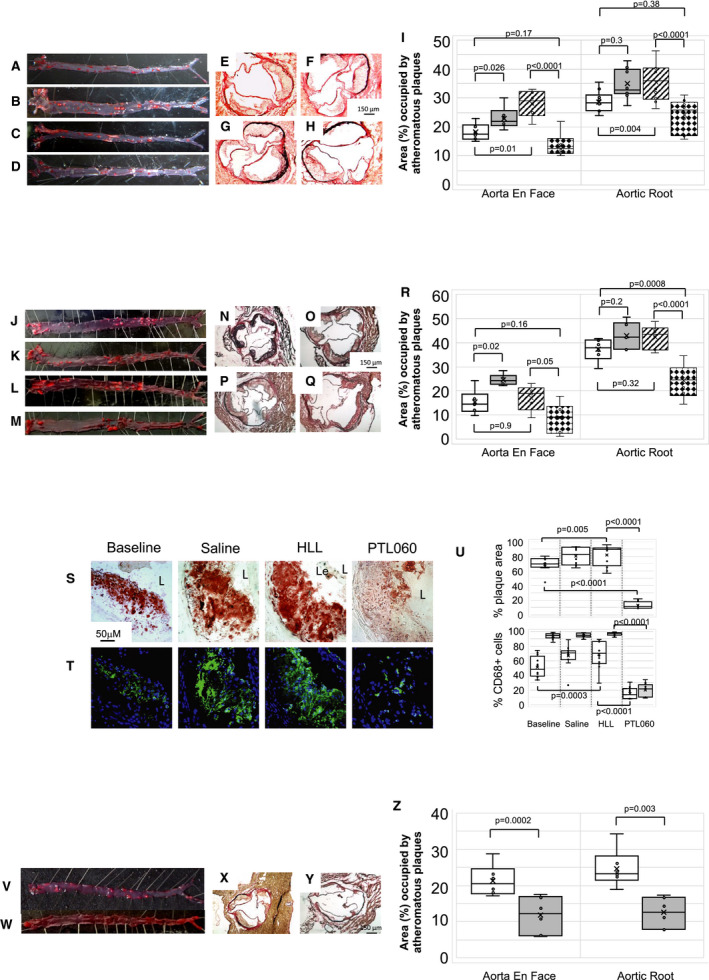
Intravenous PTL060 causes regression of atherosclerosis. **A** through **D**, Representative Oil Red O‐stained en face preparations of aorta from ApoE−/− mice fed a high‐fat diet (HFD) from age of 6 to 22 weeks (baseline: **A**), or 6 to 28 weeks with weekly injections (weeks 23–28) of saline (**B**), control cytotopic “tail” compound (**C**) or PTL060 10 μg/g (**D**). **E** through **H**, Representative light photomicrographs of elastic/van Gieson stained sections from aortic root of ApoE−/− mice fed an HFD from age of 6 to 22 weeks (baseline: **E**), or 6 to 28 weeks with weekly injections (weeks 23–28) of saline (**F**), control cytotopic “tail” compound (**G**) or PTL060 10 μg/g (**E**). **I**, Quantitative comparison of impact of PTL060 on atheroma formation in mice on HFD aged 6 to 22 weeks (white bars) or 6 to 28 weeks with weekly injections (weeks 23–28) of saline (grey bars), control “tail” compound (striped bars) or PTL060 10 μg/g (diamond bars). Comparisons of plaque development analyzed by repeated measures 2‐way ANOVA. Because multiple comparisons were made from these animals, *P*<0.008 is statistically significant. **J** through **M**, Representative Oil Red O‐stained en face preparations of aorta from ApoE−/− mice fed an HFD from age of 6 to 22 weeks (Baseline: **J**), or 6 to 28 weeks with weekly injections (weeks 23–28) of saline (**K**), control “untailed” chemically modified hirulog analogue HLL 5 μg/g (**L**) or PTL060 10 μg/g (**M**). **N** through **Q**, Representative light photomicrographs of elastic/van Gieson stained sections from aortic root of ApoE−/− mice fed an HFD from age of 6 to 22 weeks (**N**), or 6 to 28 weeks with weekly injections (weeks 23–28) of saline (**O**), control untailed chemically modified hirulog analogue HLL 5 μg/g (**P**) or PTL060 10 μg/g (**Q**). **R**, Quantitative comparison of impact of PTL060 on atheroma formation in mice on HFD aged 6 to 22 weeks (white bars) followed by weekly injections, for 6 weeks of saline (grey bars), control untailed chemically modified hirulog analogue HLL 5 μg/g (striped bars) or PTL060 10 μg/g (diamond bars). Comparisons of plaque development analyzed by repeated measures 2‐way ANOVA. Because multiple comparisons were made from these animals, *P*<0.0026 is statistically significant. **S** and **T**, Impact of PTL060 on foam cells in atherosclerosis. Representative light photomicrographs of elastic/van Gieson stained sections from aortic root (**S**) with consecutive sections analyzed by 2‐color immunofluorescence (**T**) stained with DAPI (blue) or anti‐CD68 (green). ApoE−/− mice were fed an HFD from age of 6 to 22 weeks, followed by weekly injections, for 6 weeks of saline, control untailed chemically modified hirulog analogue HLL 5 μg/g or PTL060 10 μg/g as indicated. **In the panels in S, L= lumen. Le= aortic leaflet. U**, Graphical representations of the % of plaque area staining with Oil Red O (upper panel) and, in lower panel, the percentage of area occupied by CD68+ cells (white bars) with the proportion of those CD68+ cells co‐localizing with lipid (grey bars). Each graph is a box plot with median with interquartile range with whiskers showing upper and lower limits and outliers indicated as single data points. Means are represented with “x”. Data are derived from an assessment of each of the 3 aortic root plaques from 6 individual mice, from consecutive sections as illustrated in (**S** and **T**). Comparisons of plaque development analyzed by repeated measures 2‐way ANOVA. Because multiple comparisons were made from these animals, *P*<0.0026 is statistically significant. **V** and **W**, Representative Oil Red O‐stained en face preparations of aorta from ApoE−/− mice fed a normal chow diet to the age of 28 weeks, followed by weekly injections, for 6 weeks of saline (**V**) or PTL060 10 μg/g (**W**). **X** and **Y**, Representative light photomicrographs of elastic/van Gieson stained sections from aortic root of ApoE−/− mice fed a chow diet age to the age of 28 weeks, followed by weekly injections, for 6 weeks of saline (**X**) or PTL060 10 μg/g (**Y**). **Z**, Quantitative comparison of impact of PTL060 on atheroma formation in mice on chow diet to for 28 weeks followed by weekly injections, for 6 weeks of saline (white bars) or PTL060 10 μg/g (grey bars). Comparisons of plaque development analyzed by 2 way ANOVA. *P*<0.05 is statistically significant.

To assess the impact of PTL060 on established atheroma, 6‐week old ApoE−/− mice were fed an HFD until the age of 22 weeks, before receiving intravenous injections of saline, HLL, control cytotopic tail compound or PTL060, weekly for a further 6 weeks. PTL060 caused a reduction in atheroma burden, when measured either by en face analysis or by cross‐sectional analysis of the aortic root (Figure 4A through 4R), an effect not seen after weekly injections of any of the controls, including HLL at equimolar doses (Figure 4A through 4R). This was associated with a reduction in the area of plaque occupied by lipids, as shown by Oil‐red O staining (Figure 4S and 4U), as well as a reduction in the CD68+ cells co‐localizing with lipid (Figure 4T and 4U), indicating a significant reduction in the number of foam cells within the plaques. This effect of PTL060 was evident even compared with baseline mice analyzed at week 16 before any treatment, indicating that disease regression was induced by PTL060. All the effects of PTL060 were seen without any discernible impact on body mass or circulating lipid concentrations (Table).

**Table 1:** Effect of PTL060 on Body Mass and Plasma Lipids in ApoE−/− Mice

Prevention Experiments	Aortic Tx Recipients^a^—Fed HFD 6 to 14 wk (n=6 per group)						Single Injection^a^—Fed HFD 6 to 12 wk (n=6 per group)					
	BL/6	CD31‐TFPI‐Tg	*P* Value^b^	BL/6	CD31‐Hir‐Tg	*P* Value^b^	PBS	HLL (5 μg/g)	PTL060 (2.5 μg/g)	PTL060 (5 μg/g)	PTL060 (10 μg/g)	*P* Value^c^
Body mass												
Age 6 wk	18.6±0.27	18.6±0.25	0.99	19.6±0.42	19.6±0.31	0.99	19.0±0.40	19.4±0.22	19.4±0.44	19.4±0.38	19.2±0.24	0.87
End of experiment	25.2±1.71	24.6±1.36	0.63	26.8±0.34	25.9±0.77	0.99	32.0±1.63	32.0±0.99	31.4±0.84	29.8±0.35	29.4±0.73	0.36
Cholesterol, mmol/L	53.1±3.31	52.9±3.67	0.99	50.1±4.98	50.2±5.77	0.99	51.0±5.56	54.1±5.70	49.3±4.56	50.3±4.36	50.1±5.44	0.97
Triglycerides, mmol/)	2.1±0.14	2.0±0.20	0.99	2.0±0.13	2.0±0.14	0.99	2.1±0.31	2.1±0.36	2.2±0.28	2.2±0.27	2.1±0.30	1.00
HDL, mmol/L	1.5±0.11	1.5±0.10	0.99	1.6±0.07	1.5±0.08	0.99	1.4±0.28	1.5±0.08	1.6±0.12	1.5±0.09	1.5±0.09	0.92
LDL, mmol/L	57.2±2.73	55.2±3.49	0.97	63.9±9.97	61.0±6.28	0.92	55.6±6.64	54.7±4.48	54.1±4.72	51.7±5.89	52.8±5.42	0.99

Regression Experiments	6‐wk‐old	Series 1 HFD 6 to 28 wks (n=6 per group)					Series 2 HFD 6 to 28 wk (n=6 per group)					
		Baseline^d^	PBS^e^	Tail Only	PTL060 (10 μg/g)	*P* Value^c^	Baseline^d^	PBS	HLL (5 μg/g)	PTL060 (5 μg/g)	PTL060 (10 μg/g)	*P* Value^c^
Body mass												
Age 6 wk	···	19.3±0.28	19.6±0.37	19.1±0.15	19.3±0.38	0.74	20.2±0.39	20.1±0.38	20.6±0.07	20.1±0.30	20.3±0.26	0.73
Age 22 wk	···	30.5±0.58	32.8±0.44	30.4±0.90	32.0±0.81	0.19	31.5±1.34	31.4±0.84	31.1±0.66	31.2±1.00	31.9±0.78	0.85
End of experiment	···	···	33.2±0.31	31.8±0.73	28.7±1.55	0.03	···	32.9±1.19	31.4±0.33	30.2±0.93	29.8±0.59	0.11
Cholesterol, mmol/L	10.3±4.6	56.7±6.08	61.5±6.51	61.9±6.35	57.9±3.61	0.88	54.9±6.25	56.9±6.93	54.1±5.39	53.9±6.31	54.3±13.1	0.41
Triglycerides, mmol/L	0.36±0.14	2.08±0.15	2.25±0.11	2.49±0.13	2.19±0.16	0.26	2.27±0.28	1.68±0.31	2.21±0.38	2.2±0.33	2.16±0.69	0.86
HDL, mmol/L	4.46±1.1	1.54±0.007	1.49±0.06	1.46±0.06	1.56±0.06	0.65	1.61±0.12	1.58±0.11	1.78±0.2	1.6±0.16	1.68±0.38	0.27
LDL, mmol/L	14.4±2.8	46.5±3.39	56.1±2.92	57.4±2.57	50.8±2.84	0.07	53.8±3.51	62.7±4.43	63.5±3.55	60.6±6.79	63.3±16.7	0.97

“n” refers to number of animals per group. Samples from each animal were analyzed in triplicate. BL/6 indicates C57BL/6J; HDL, high‐density lipoprotein; HFD, high‐fat diet; HLL, hirulo analogue chemically modified to accept the myristoyl tail (Please note: HLL 5 μg is equimolar to PTL060 10 μg); LDL, low‐density lipoprotein; and TFPI, tissue factor pathway inhibitor.

a In prevention experiments, aortic transplants performed, and single injections given to mice aged 8 weeks, 2 weeks after starting a high‐fat diet.

b Two‐way ANOVA for multiple groups.

c One‐way ANOVA for multiple groups (NB: values from 6‐week‐old mice not included in comparisons).

d Baseline=week 22. Mice in the “baseline” groups were harvested at this timepoint before any treatment.

e Treatments given weekly by intravenous injection for 6 weeks (mice aged 22–28 weeks).

PTL060 also significantly reduced atheroma burden after administration to ApoE−/− mice fed a normal chow diet, weekly from the age of 28 weeks for 6 weeks (Figure 4V through 4Z). Under both HFD and chow dietary conditions, administration of PTL060 was accompanied by significant reductions in plasma levels of TNFα, IFNγ, MIF, and CCL2 (Figure S4), compared with the appropriate controls.

### Phenotype of Regressing Plaques After PTL060 Treatment

**Figure 5:**
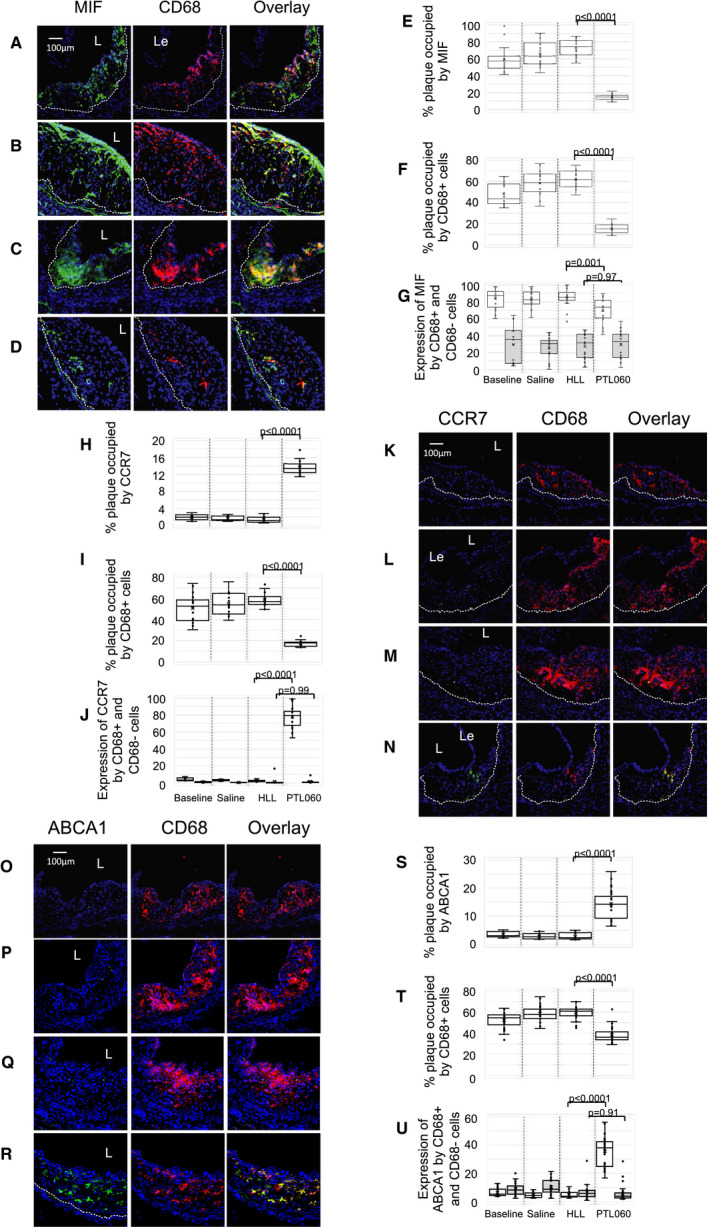
Phenotype of plaque cells after PTL060. Three color immunofluorescence images show confocal microscopic analysis of consecutive sections of aortic roots of ApoE−/− mice, fed a high‐fat diet from 6 to 22 weeks (“Baseline” **A**,** K**, and **O**) or 6 to 28 weeks, with mice administered weekly injections of saline (**B**,** L**, and **P**), chemically modified hirulog analogue HLL (**C**,** M**, and **Q**), or PTL060 (**D**,** N**, and **R**) as indicated between weeks 22 to 28. Panels show the plaque expression of CD68 (red) with (green) either macrophage migration inhibitory factor (**A** through **D**) CCR7 (**K** through **N**) or ATP‐binding cassette transporter – 1 (**O** through **R**). Yellow in overlay image indicates co‐localization. The plaque area is demarcated by the lumen (**L**) and the dotted white line. Le=aortic leaflet. Each panel of images is accompanied by graphical representations of the percentage of plaque area staining for the molecule of interest (**E**‐Macrophage migration inhibitory factor , **H**‐CCR7, **S**‐ATP‐binding cassette transporter – 1) and the percentage of plaque area occupied by CD68+ (**F**,** I**, and **T**) and the proportion of CD68+ cells (white bars) and CD68‐negative cells (grey bars) co‐staining for macrophage migration inhibitory factor (**G**), CCR7 (**J**), or ATP‐binding cassette transporter – 1 (**U**). Each graph is a box plot with median with interquartile range with whiskers showing upper and lower limits and outliers indicated as single data points. Means are represented with “x”. Each is derived from an assessment of each of the 3 aortic root plaques from 6 to 24 individual mice. Comparisons of plaque composition analyzed by repeated measures 2‐way ANOVA. Because multiple comparisons were made from these animals, *P*<0.0026 is statistically significant. ABCA1 indicates ATP‐binding cassette transporter – 1; CCR7, C‐C chemokine receptor type 7; and MIF, macrophage migration inhibitory factor.

Atheromatous plaques in ApoE−/− mice fed an HFD from 6 to 22 weeks of age (baseline) contained a significant number of CD68+ cells (monocytes/macrophages), occupying ≈45% of plaque area (Figure 5A and 5F). Compared with control animals injected with either saline or HLL (Figure 5B through 5G), weekly injections of PTL060 reduced the proportion of plaque area occupied by CD68+ cells to below 20% (Figure 5D and 5F) with an associated increase in the proportion of plaque cells that were CD68‐negative (Figure 5G). The proportion of plaque area staining for MIF reduced from >60% at baseline (Figure 5A and 5E) to <20% (Figure 5D and 5E) though the proportion of CD68+ and CD68‐negative cells that expressed MIF was not altered (Figure 5F and 5G). These data indicate that PTL060 induced a shift in plaque cell composition, from predominantly CD68+ cells in control mice to predominantly CD68‐negative cells after PTL060 treatment, in association with a marked reduction in vessel wall MIF expression.

Plaques developing in mice fed an HFD between 6 and 22 weeks were almost devoid of cells expressing the chemokine receptor CCR7 (C‐C chemokine receptor type 7) (Figure 5H and 5K) or the cholesterol efflux regulator ATP‐binding cassette transporter molecule ABCA1 (Figure 5O and 5S), with <10% of plaque cells co‐expressing these molecules (Figure 5I, 5J, 5T, and 5U). Six weeks of treatment with weekly PTL060 from weeks 22 to 28 caused significant increases in the proportion of plaque area occupied by cells expressing CCR7 and ABCA1 (Figure 5H, 5N, 5R, and 5S) and almost all of these were CD68+ cells (Figure 5I, 5N, 5R, and 5T), compared with control saline‐treated mice and in contrast to mice treated with weekly injections of HLL, in whom expression of both CCR7 and ABCA1 was not statistically different to that seen at baseline (Figure 5H through 5M and 5O through 5U).

In parallel, little interleukin‐10 staining was seen within the plaques of any of the control animals (Figure S5A and S5B), and <2% of plaque‐infiltrating CD68+ cells co‐expressed interleukin‐10 (Figure S5C), whereas almost all plaque infiltrating CD68+ cells expressed IFNγ (Figure S5E and S5G) and TNFα (Figure S5I and S5K) and 15% to 20% of plaque CD68‐negative cells expressed these 2 proinflammatory cytokines (Figure S5H and S5L). After 6 weeks of PTL060, 10% to 15% of plaque area stained for interleukin‐10 (Figure S5A and S5B), including 60% of the plaque‐infiltrating CD68+ cells (Figure S5C), and ≈10% of CD68‐negative cells (Figure S5D). There was a marked reduction in both the proportion of plaque area (Figure S5E, S5F, S5I, and S5J) and the proportion of CD68+ cells (Figure S5G and S5K) staining for INFγ and TNFα, compared with control saline‐treated mice and in contrast to mice treated with weekly injections of HLL, in whom no reductions were seen. Similar dichotomous patterns of staining were seen for the macrophage polarization markers iNOS (Figure S6A through S6D) and CD206 (Figure S6E through S6H), with staining for the former suppressed in both CD68+ and CD68‐negative cells, but staining of the latter enhanced in CD68+ cells, by weekly injections of PTL060.

Thus, 6 weeks of weekly PTL060 injections promoted a significant reduction in plaque CD68+ cells and a significant shift in their phenotype, towards a phenotype that has previously been associated with plaque regression (CCR7+, ABCA1+, IFNγ−, interleukin‐10+, iNOS−, CD206+).

### Mechanism of Regression: Impact of Thrombin Inhibitor Tethered to the Surface of Circulating Monocytes

As shown already, PTL060 rapidly adheres to the surface of circulating leukocytes. To investigate the impact of this leukocyte‐tethered thrombin inhibitor, in isolation to that tethered by EC or platelets and erythrocytes, we adoptively transferred CD11b+ cells labeled with the fluorescent dye PKH26 into ApoE−/− mice fed an HFD from weeks 6 to 22, before assessing the phenotype of the labeled plaque cells by confocal immunofluorescence microscopy 48 hours later. To avoid the potential confounding influence of transfer of PTL060 from the adoptively transferred cells to vascular membranes, for these experiments we used CD11b+ cells from CD31‐Hir‐Tg mice, which express covalently tethered cell surface hirudin on all monocytes, and compared the impact of labeled BL/6 cells.

**Figure 6:**
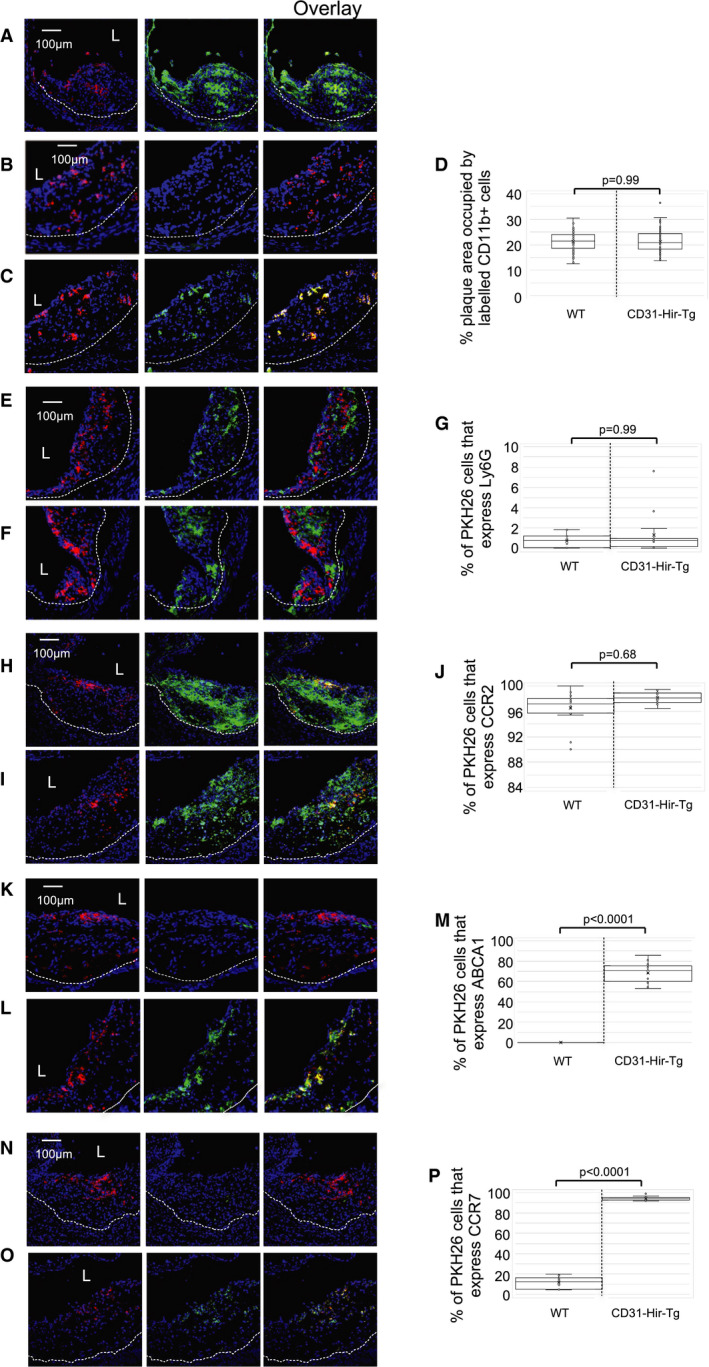
Impact of adoptive transfer of CD11b+ cells expressing tethered thrombin inhibitor. All panels: CD11b cells, harvested from either BL/6 or CD31‐Hir‐Tg mice were labeled in vitro with PKH26 (red) and adoptively transferred into ApoE−/− mice fed a high‐fat diet between ages of 6 to 22 weeks. Aortic roots were collected 48‐hours post‐injection, for confocal immunofluorescence analysis of the phenotype of adoptively transferred cells. Graphs are a box plot with median with interquartile range with whiskers showing upper and lower limits and outliers indicated as single data points. Means are represented with “x”. Each is derived from an assessment of at least 3 aortic root plaques from 6 to 35 individual mice. **A**, To illustrate the expression of macrophage migration inhibitory factor (green) at baseline age 22 weeks, throughout the plaque area in a mouse that received BL/6 CD11b+ cells. **B** through **D**, Comparison of the recruitment of CD11b+ cells from BL/6 (**B**) and CD31‐Hir‐Tg (**C**) mice. Hirudin (green) only seen in cells from CD31‐Hir‐Tg mice. **D**, Illustrates quantitative assessment of the proportion of plaque area occupied by PKH26+ cells. **E** through **G**, To illustrate expression of Ly6G (green) within the plaque after adoptive transfer of CD11b+ cells from BL/6 (**E**) or CD31‐HIr‐Tg (**F**) mice. **G**, Illustrates quantitative assessment of the proportion of PKH26+ cells co‐expressing Ly6G. **I** and **J**, To illustrate expression of CCR2 (green) within the plaque after adoptive transfer of CD11b+ cells from BL/6 (**H**) or CD31‐HIr‐Tg (**I**) mice. **J**, Illustrates quantitative assessment of the proportion of PKH26+ cells co‐expressing CCR2. **K** through **M**, To illustrate expression of ATP‐binding cassette transporter – 1 (green) within the plaque after adoptive transfer of CD11b+ cells from BL/6 (**K**) or CD31‐HIr‐Tg (**L**) mice. M illustrates quantitative assessment of the proportion of PKH26+ cells co‐expressing ATP‐binding cassette transporter – 1. **N** through **P**, To illustrate expression of CCR7 (green) within the plaque after adoptive transfer of CD11b+ cells from BL/6 (**N**) or CD31‐HIr‐Tg (**O**) mice. **P**, Illustrates quantitative assessment of the proportion of PKH26+ cells co‐expressing CCR7. Quantitative comparisons analyzed by repeated measures 2‐way ANOVA. Because multiple comparisons were made from these animals, *P*<0.0055 is statistically significant. ABCA1 indicates ATP‐binding cassette transporter – 1; CCR2, C‐C chemokine receptor type 2; CCR7, C‐C chemokine receptor type 7; and WT, wild‐type

At the point of adoptive transfer, MIF was expressed throughout the plaques (Figure 6A), and significant numbers of labeled cells were recruited, such that they occupied 20% to 25% of plaque area (Figure 6B and 6C), with no difference in the numbers of BL/6 versus CD31‐Hir‐Tg cells recruited (Figure 6D). All recruited cells from the Tg strain expressed hirudin (Figure 6C). Although the plaques already contained significant numbers of Ly6G+ granulocytes, occupying 20% to 25% of plaque area, and although the adoptively transferred CD11b+ populations contained granulocytes, <1% of the recruited PKH26‐labeled cells co‐expressed Ly6G (Figure 6E through 6G), suggesting they were mostly monocytes, and >95% of the labeled cells, from both BL/6 and CD31‐Hir‐Tg, expressed CCR2 (Figure 6H through 6J), suggesting they were predominantly Ly6Chi monocytes. Whereas none of the recruited BL/6 cells expressed ABCA1 or CCR7, the majority of CD31‐Hir‐Tg cells recruited to the plaques expressed both these markers (Figure 6K through 6P). In addition, whereas the majority of recruited BL/6 cells expressed IFNγ and iNOS, these were expressed by few of the recruited CD31‐Hir‐Tg cells (Figure S7). Instead, a significant minority of these cells expressed interleukin‐10 and CD206, markers not expressed by labeled BL/6 cells (Figure S7). These data illustrate that CCR2+ monocytes recruited to established plaques are polarized towards a proinflammatory M1 phenotype, but that a membrane tethered anti‐thrombin subverts this phenotype towards one that has previously been associated with plaque regression. This strongly suggests that the shift towards regression, induced by PTL060, begins immediately post‐injection via the influence of cell tethered PTL060 on the phenotype of CCR2+ monocytes recruited to existing plaques.

### Monocyte Recruitment and Phenotype After Systemic PTL060

**Figure 7:**
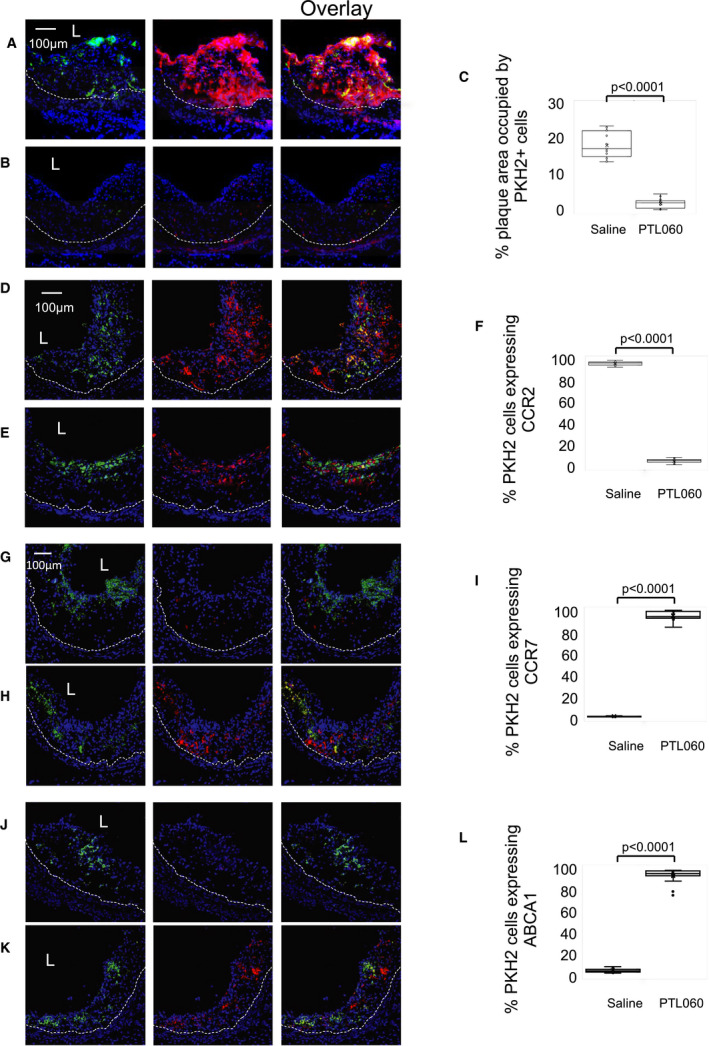
Monocyte recruitment and phenotype after systemic PTL060. Confocal microscopic analysis of 3‐color immunofluorescence images through consecutive sections of aortic roots of ApoE−/− mice, fed a high‐fat diet from 6 to 26 weeks, with mice administered weekly injections of saline or PTL060 as indicated below between weeks 22 to 25. One week after the last injection, mice were injected with PKH2‐labeled CD11b cells (green) and aortic roots harvested 48 hours later. Graphs are a box plot with median with interquartile range with whiskers showing upper and lower limits and outliers indicated as single data points. Means are represented with “x”. Each is derived from a double assessment of each of the 3 aortic root plaques from 3 individual mice. **A** through **C**, To illustrate the expression of macrophage migration inhibitory factor (red) after adoptive transfer of BL/6 CD11b+ cells in mice treated with saline (**A**) or PTL060 (**B**). **C**, Illustrates quantitative assessment of the proportion of plaque area occupied by PKH2+ cells. **D** through **F**, To illustrate the expression of CCR2 (red) after adoptive transfer of BL/6 CD11b+ cells in mice treated with saline (**D**) or PTL060 (**E**). **F**, Illustrates quantitative assessment of the proportion of PKH2+ cells co‐expressing CCR2. **G** through **I**, To illustrate the expression of CCR7 (red) after adoptive transfer of BL/6 CD11b+ cells in mice treated with saline (**G**) or PTL060 (**H**). **I**, Illustrates quantitative assessment of the proportion of PKH2+ cells co‐expressing CCR7. **J** and **K**, To illustrate the expression of ATP‐binding cassette transporter – 1 (red) after adoptive transfer of BL/6 CD11b+ cells in mice treated with saline (**J**) or PTL060 (**K**). **L**, Illustrates quantitative assessment of the proportion of PKH2+ cells co‐expressing ATP‐binding cassette transporter – 1. Quantitative comparisons analyzed by repeated measures 2‐way ANOVA. Because multiple comparisons were made from these animals, *P*<0.007 is statistically significant. ABCA1 indicates ATP‐binding cassette transporter – 1; CCR2, C‐C chemokine receptor type 2; and CCR7, C‐C chemokine receptor type 7.

To assess whether PTL060 reduced numbers of monocytes recruited, ApoE−/− mice were fed an HFD from 6 to 22 weeks, before administration of weekly PTL060 or saline for 3 weeks to the age of 25 weeks. PKH2‐labeled CD11b cells from BL/6 mice were administered 1 week after the last injection of PTL060 (by which time all PTL060 should have left the circulation [see Figure 2]), and plaques examined 48 hours later by confocal immunofluorescence microscopy. After 3 weeks treatment with PTL060, there was significant suppression of MIF expression by vessel wall cells (Figure 7A and 7B), associated with a significant reduction in the number of adoptively transferred cells recruited to the plaques, such that they occupied only 2% of plaque area compared with 20% in control mice that had received saline (Figure 7C).

As at baseline, the monocytes recruited into plaques of saline treated animals were predominantly CCR2+(Figure 7D and 7F), suggesting they belonged to the Ly6Chi subset. However, monocytes recruited to plaques in PTL060‐treated mice were predominantly CCR2‐negative, suggesting they were predominantly Ly6Clo monocytes (Figure 7E and 7F). Compared with cells recruited in saline treated mice, the majority of labeled cells recruited into the plaques of PTL060 mice expressed CCR7 (Figure 7G through 7I) and ABCA1 (Figure 7J through 7L), as well as interleukin‐10 (Figure S8A through S8C) but fewer cells expressed TNFα (Figure S8D through S8F) or IFNγ (Figure S8G through S8I). Therefore, weekly systemic delivery of PTL060 suppressed vessel wall chemokine production, significantly reduced the recruitment of adoptively transferred monocytes by >90% compared with controls and promoted recruitment of CCR2‐negative monocytes, which, independently of any direct binding of PTL060 to their cell surface, had the same phenotype that has already been associated with plaque regression.

### Thrombin Inhibitor on the Surface of CD11b+ Cells is Sufficient to Induce Regression

To assess whether tethering of PTL060 to leukocytes alone was sufficient to induce plaque regression, we fed ApoE−/− mice an HFD from 6 to 22 weeks, and then adoptively transferred, by weekly intravenous injection during weeks 23 to 28, CD11b+ cells, while continuing the HFD. Control mice received cells from BL/6 mice incubated, before transfer, with either saline or the cytotopic tail compound only. Experimental mice received BL/6 cells pre‐incubated with PTL060 or, as a positive control, cells from CD31‐Hir‐Tg mice. The 30‐minute incubation with these compounds had minimal effect on the viability of adoptively transferred cells (Table S2).

**Figure 8:**
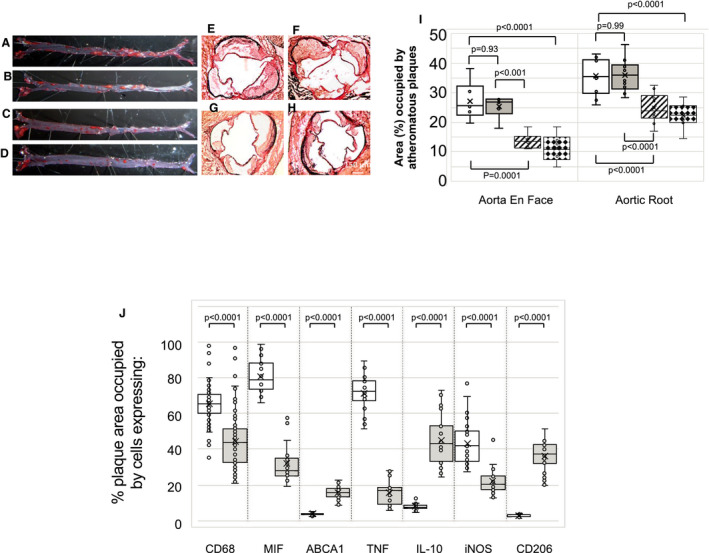
Regression induced by thrombin inhibitor on isolated CD11b+ cells. Samples represented here are from ApoE−/− mice fed a high‐fat diet from age of 6 to 28 weeks with weekly (weeks 23–28) injections of CD11b+ cells from BL/6 mice pre‐incubated with saline (**A** and **E**), control “tail” molecule (**B** and **F**), PTL060 100 μmol/L (**C** and **G**) or with CD11b cells from CD31‐Hir‐Tg mice (**D** and **H**). **A** through **D**, Representative Oil Red O‐stained en face preparations of aorta. **E** through **H**, Representative light photomicrographs of elastic/van Gieson stained sections from aortic root. **I**, Quantitative comparison of atheroma regression in the whole aorta (en face) or aortic root of mice fed a high‐fat diet from age of 6 to 28 weeks with weekly (weeks 23–28) injections of CD11b+ cells from BL/6 mice pre‐incubated with saline (white bars), control “tail” molecule (grey bars), PTL060 100 μmol/L (striped bars) or with CD11b cells from CD31‐Hir‐Tg mice (diamond bars). **J**, Graph is a box plot with median with interquartile range with whiskers showing upper and lower limits and outliers indicated as single data points. Means are represented with “x”. Each is derived from an assessment of at least 3 aortic root plaques from 6 to 24 individual mice. It illustrates the proportion of plaque area occupied by cells expressing the various markers (as indicated on abscissa) from mice receiving CD11b+ cells from BL/6 mice pre‐incubated with saline (white bars) or CD31‐Hir‐Tg mice (grey bars). Quantitative comparisons in (**I** and **J**) analyzed by repeated measures 2‐way ANOVA. Because multiple comparisons were made from these animals, *P*<0.0055 is statistically significant. ABCA1 indicates ATP‐binding cassette transporter – 1; IL‐10, interleukin‐10; iNOS, inducible NO synthase; MIF, macrophage migration inhibitory factor; and TNF, tumor necrosis factor.

All mice receiving control cells showed progression of atherosclerosis between 23 and 28 weeks (Figure 8A, 8B, 8E, 8F, and 8I) that was not statistically different in degree to that seen in saline treated controls described earlier (see Figure 4). In contrast, mice receiving PTL060‐treated BL/6 cells (Figure 8C, 8G, and 8I), or cells from CD31‐Hir‐Tg mice (Figure 8D, 8H, and 8I) showed regression of plaque area not statistically different in degree to mice that had been treated with systemic PTL060 (see Figure 4). The phenotype of regressing plaques in mice given cells from CD31‐Hir‐Tg mice strongly resembled those in mice receiving systemic PTL060 (Figure 8J).

Taken together with the data from the adoptive transfer of labeled cells, these data indicate that mechanistically, the impact of systemic PTL060 treatment can be reproduced entirely by isolating a thrombin inhibitor onto the surface of circulating monocytes, suggesting that inhibiting thrombin activity on only these cells is sufficient to promote regression.

## Discussion

The involvement of coagulation proteases in atherosclerosis and the impact of inhibiting them has been described by multiple groups in previous studies. For instance, ApoE^−/−^ mice made deficient in HCII, a natural thrombin inhibitor,^17^ or carrying a DNA variant resulting in defective thrombomodulin‐mediated generation of activated protein C^18^ develop severe atheroma, indicating that in this model, endogenous regulators of thrombin act to limit disease severity. Conversely, factor Xa inhibitors^19^, ^20^ and direct thrombin inhibitors^21^, ^22^, ^23^, ^24^ prevent atheroma progression and maintain plaque stability. Systemic anticoagulants can also induce regression of atherosclerosis in ApoE−/− mice. Bea et al used melagatran in 30‐week‐old animals and showed reduced burden of advanced atheromatous lesions associated with plaque stability.^25^ More recently, Posthuma et al^26^ reduced atheroma burden in 22‐week‐old animals by 25% after daily treatment for 6 weeks with clinically relevant doses of the factor Xa inhibitor rivaroxaban.

These data from animal models have fed into clinical practice, and the benefit of systemic anticoagulation in patients with atherosclerosis has been most recently confirmed by the COMPASS (Cardiovascular Outcomes for People using Anticoagulation Strategies) trial, which showed that addition of rivaroxaban to aspirin in patients with stable atherosclerotic cardiovascular disease led to fewer deaths, strokes, and myocardial infarction.^27^ Moreover, the PAR‐1 antagonist vorapaxar has also been shown to reduce the risk of myocardial infarction in patients with stable atherosclerosis.^28^ However, these benefits were associated with a significant increase in the incidence of major bleeding events; this is the biggest drawback to using systemic anticoagulants or antiplatelet drugs for non‐thrombotic diseases, as their impact on hemostasis cannot be separated from their clinical efficacy.

The development of thrombalexins built upon a foundation of tethering anti‐complement compounds using a generic tail based on the myristoyl‐electrostatic switch.^29^, ^30^ We have demonstrated that several versions of thrombalexin, including PTL060 effectively bind to cell membranes, maintain potent thrombin inhibitory activity, and prevent intravascular thrombosis when infused into rodent^12^ or primate kidneys^14^ before transplantation. Under these circumstances, PTL060 remains detectable in tissue for several days.

In this work, we have shown that after intravenous injection, PTL060 inhibits secretion of vessel wall chemokines for 1 week and prevents atheroma formation but increases the risk of bleeding for only 24 hours. Therefore, the addition of the cytotopic tail uncouples the pharmacodynamics of hirulog's effects on hemostasis from its effects on atheroma formation, so that an increased bleeding tendency is seen for only one seventh of the period between doses that both prevent plaque formation and induce plaque regression. To our knowledge, this is the first demonstration of such uncoupling, and represents a significant advance in understanding the true therapeutic potential of targeting coagulation proteases to influence inflammatory disease.

Our interest in this area began with the idea that targeting anticoagulants to cell membranes would achieve high concentrations in localized environments, such as the endothelium of an organ transplant, to inhibit vascular thrombosis. We demonstrated proof of concept using transgenically expressed fusion proteins,^15^, ^31^ and in the process showed that inhibiting thrombin‐mediated signaling through PARs on vessels inhibited local chemokine gradients, which reduced monocyte recruitment to sites of inflammation, including after transplantation, and prolonged survival.^32^ We then went on to show that thrombin was similarly involved in chemokine gradient generation in atherosclerosis, such that expression of a tethered anticoagulant on SMC significantly reduced the development of atheroma in ApoE−/− mice.^11^ In this new work we have confirmed that expression of tethered anticoagulants on EC is equally efficacious at suppressing vessel wall chemokine expression by both EC and SMC in ApoE−/− mice and equally effective at preventing atherosclerosis as expression on SMC. Although there was some variation in the extent of atherosclerosis development by control ApoE−/− mice fed an HFD for 4 to 6 weeks across temporally distinct experiments, one consistent feature was that single doses of PTL060 caused significant inhibition (≥50%) of atheroma formation compared with controls. We have not investigated the mechanism by which targeted thrombin inhibition on EC influences the phenotype of underlying SMC, but the data are consistent with the known importance of EC/SMC interplay for atheroma development,^33^ and one possibility is that it acts via regulation of angiopoietin‐2 secretion, known to be important in atherosclerosis,^34^ which we have shown to be thrombin‐dependent in a separate model system.^35^

Our most important finding was that in ApoE−/− mice fed an HFD for 16 weeks before weekly injections of PTL060 for 6 weeks, atheroma burden was significantly reduced, compared not only to control mice given either saline or an equimolar dose of parental HLL, but also in comparison with baseline, indicating that PTL060 caused regression of existing disease. This was achieved without impacting plasma lipid concentrations. A similar reduction in plaque burden was seen in mice fed a normal chow diet. There were significantly fewer CD68+ macrophages and foam cells present after 6 weeks treatment in the regressing plaques.

In assessing the mechanisms of regression, we considered the importance of inhibiting vessel wall chemokine gradients. Continuous monocyte recruitment into the vessel wall is one of the major steps in the pathogenesis of atherosclerosis, as evidenced by studies showing that simultaneous inhibition of CCL2, CX3CR1 and CCR5 near abolishes development of atheroma in ApoE−/− mice.^36^ In addition, deficiency of MIF also impairs atheroma development in low‐density lipoprotein‐receptor deficient mice^37^ an inhibitory anti‐MIF antibody has been shown to prevent atherosclerosis in ApoE^−/−^ mice,^38^ and our previous work illustrated that MIF secretion was important. We confirmed that a single dose of PTL060 led to prolonged suppression of vessel wall MIF (and CCL‐2), and that this associated with prevention of plaque development. In addition, recruitment of labeled CD11b+Ly6G‐ monocytes, adoptively transferred 1 week after the last of 3 doses of PTL060, was reduced by 90%, compared with that seen in control, saline‐treated mice. This is consistent with the idea that suppression of vessel wall chemokine expression, interrupting the continuous cycle of monocyte recruitment, foam cell development, cell death, and vessel wall inflammation might be an important contributory mechanism of how PTL060 induces plaque regression.

However, PTL060 also modulated the phenotype of recruited monocytes/macrophages. Thus, plaque cells in the regressing plaques in PTL060‐treated mice had a significantly different phenotype compared with those detected in the progressing plaques in control animals, with reduced expression of proinflammatory IFNγ, TNFα, and iNOS, and significant increases in the proportions of cells expressing CD206, interleukin‐10, ATP‐binding cassette transporter – 1, and CCR7.

These phenotypic characteristics have all been associated with mechanisms of regression defined in other studies. For instance after transplantation of atheromatous aorta from ApoE−/− mice into BL/6 mice,^39^, ^40^, ^41^ the chemokine receptor CCR7 was shown to be important for emigration of foam cells, as demonstrated by inhibiting the chemokine ligands for CCR7.^42^ In another model, low‐density lipoprotein‐receptor‐deficient mice treated with an antisense to micro‐RNA‐33 showed regression associated with upregulated ABCA1 expression in plaque macrophages and enhanced reverse cholesterol transport,^43^ in association with increased levels of circulating HDL, consistent with the known importance of ABCA1 for cholesterol loading into HDL and with the phenotype of ABCA1‐deficient mice.^44^ Finally, the importance of polarizing new monocyte recruits to the plaque towards an M2 phenotype has been recently demonstrated in the aortic transplant model, by confirming that regression is dependent on the expression of both appropriate chemokine receptors (CCR2/CX3CR1) and signal transducer and activator of transcription 6 by recipient monocytes.^45^

These phenotypic changes were evident in newly recruited monocytes, but our adoptive transfer experiments suggested that CCR2+ or CCR2− monocytes were recruited at different times following PTL060 treatment. In the first experimental setting, using CD11b+ cells from CD31‐Hir‐Tg mice, transferred into ApoE−/− mice fed an HFD for 16 weeks without PTL060 treatment, we showed that CCR2+ monocytes were predominantly recruited, and these displayed the phenotypic traits associated with regression. In a second experimental setting, we showed that the CD11b+ cells recruited after adoptive transfer into mice already treated with 3 doses of PTL060 were predominantly CCR2− cells but also displaying the same phenotypic traits associated with regression. In this situation, labeled cells were transferred 1 week after the last of 3 doses of PTL060, into mice in which PTL060 had been cleared from the circulation, but importantly, into mice in which significant changes in plaque phenotype had already been induced. We suggest that the differential recruitment of the CCR2− (Ly6Clo) subset, known to be precursors of M2 polarized macrophages,^46^ is most likely because of the conditions within the plaque already established by the PTL060. We postulate that, after the first dose, the immediate uptake of PTL060 onto the surface of circulating CCR2+ monocytes, protects them from thrombin as they are recruited into established plaques, significantly skews their phenotype as they become macrophages, and this rapidly establishes the microenvironmental conditions inside the plaque that are required to initiate regression.

Although the focus of this work has been on the impact of thrombin inhibition on the vessel wall and circulating leukocytes, they also provide a potential explanation for the mechanisms through which factor Xa inhibitors induce regression, though these agents would also influence signaling through PAR‐2, something we've not addressed. We also showed immediate uptake of PTL060 onto circulating platelets after intravenous injection. Since interactions between platelets and EC and between platelets and leukocytes, via CD40, have been shown to promote leukocyte recruitment and exacerbate plaque formation in this model,^47^ we cannot exclude the possibility that PTL060 might be modulating these interactions.

However, the data generated showing that weekly adoptive transfer of CD11b+ cells pre‐treated with PTL060, or expressing a transgenic hirudin fusion protein can induce the same degree of regression as systemic PTL060, suggests that protecting plaque‐recruited monocytes from the direct effects of thrombin is the key factor required for regression. Since thrombin, via PAR‐1 and cullin 3‐mediated degradation is known to promote post‐transcriptional downregulation of ABCA1 in macrophages,^48^ and is also known to promote M1 polarization of microglia after intracerebral hemorrhage,^49^ our data are most consistent with the hypothesis that thrombin plays a hitherto unrecognized but pivotal role in determining the inflammatory phenotype of plaque macrophages and promoting plaque progression.

## Sources of Funding

This work was funded by the Wellcome Trust (award 098300/Z/12/Z). Additional support was via the Medical Research Council (MR/P018513/1). We acknowledge the financial support of the King's Commercialisation Institute.

## Disclosures

RAS and AD are authors of a patent covering topical use of PTL060 in organ transplants.

## Supporting information


**Tables S1–S2Figures S1–S8**
