# Supplementary material for: Regression of Atherosclerosis in ApoE−/− Mice Via Modulation of Monocyte Recruitment and Phenotype, Induced by Weekly Dosing of a Novel “Cytotopic” Anti‐Thrombin Without Prolonged Anticoagulation
Source: J Am Heart Assoc. 2020 Jul 2;9(13):e014811. doi: 10.1161/JAHA.119.014811 (PMC7670518; doi:10.1161/JAHA.119.014811)
Supplement: Supplementary file 1 — Tables S1–S2Figures S1–S8 [file JAH3-9-e014811-s001.pdf]

Supplemental Material

Table S1.
% viability within Macrophage Forward Scatter / Side scatter gate, as assessed by LIVE /Dead aqua fluorescent dye.
See methods for details

	Incubation time (minutes)		
	30	60	120
Control PBS	-	-	99
25μM PTL060	99	95	96
50 μM PTL060100 μM	96	98	92
PTL060	97	87	80

Table S2. Viability of adoptively transferred CD11b cells, assessed by trypan blue exclusion, after incubation with saline, control tail only peptides (100μM), or PTL060 (100μM) for 30 minutes immediately prior to injection.

	Incubated with		
	Saline	Control tail peptides only	PTL060
Mean % viability	97.1	97.1	97.2
SEM	0.16	0.1	0.16

Figure S1. Illustration of the in vivo models used in this manuscript. A: aortic transplantation. B: prevention of atherosclerosis. C: Regression of atherosclerosis.

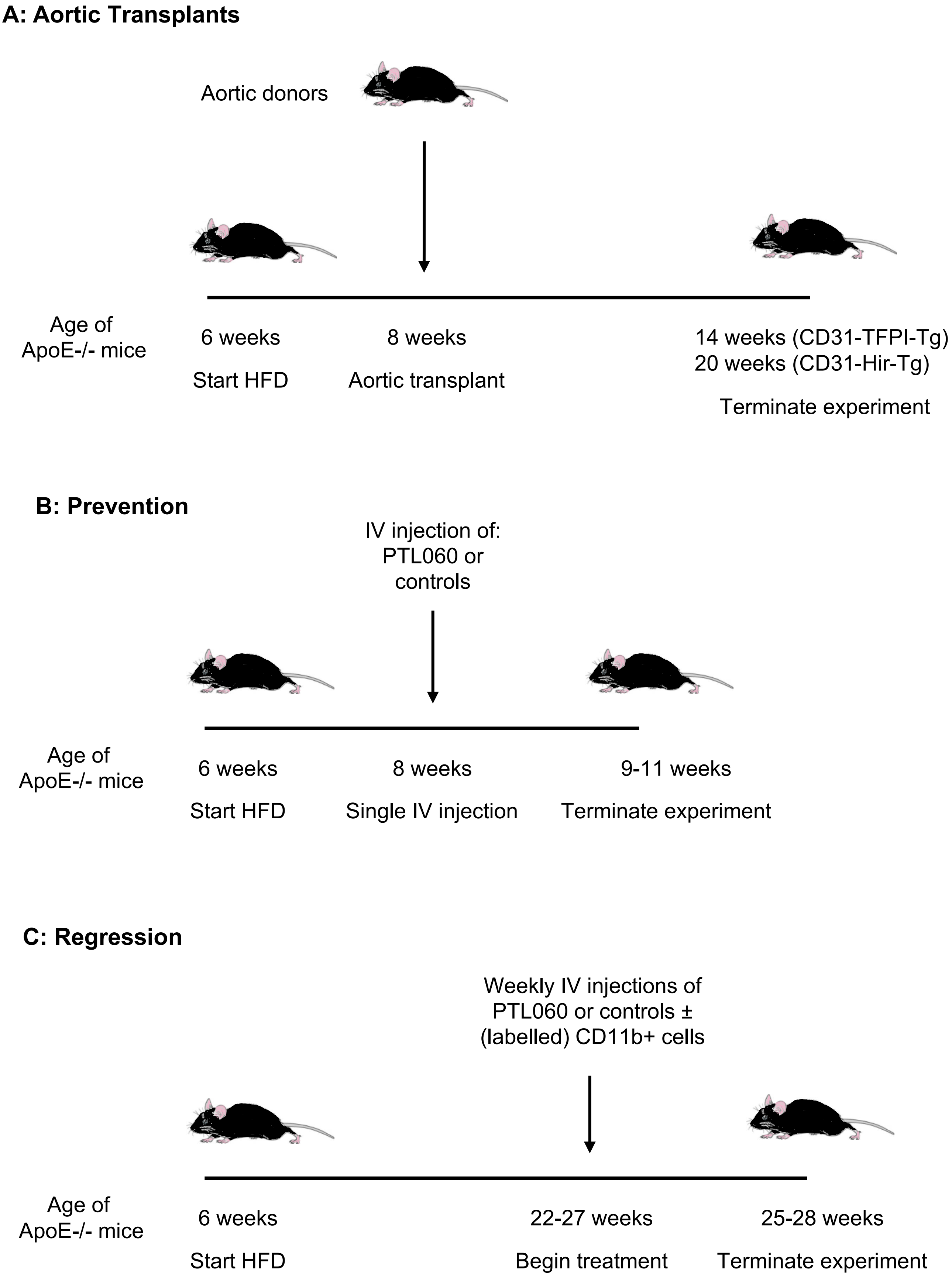

Figure S2. Inhibition of TF or thrombin on EC abolishes CCL2 and MIF expression in vascular walls.

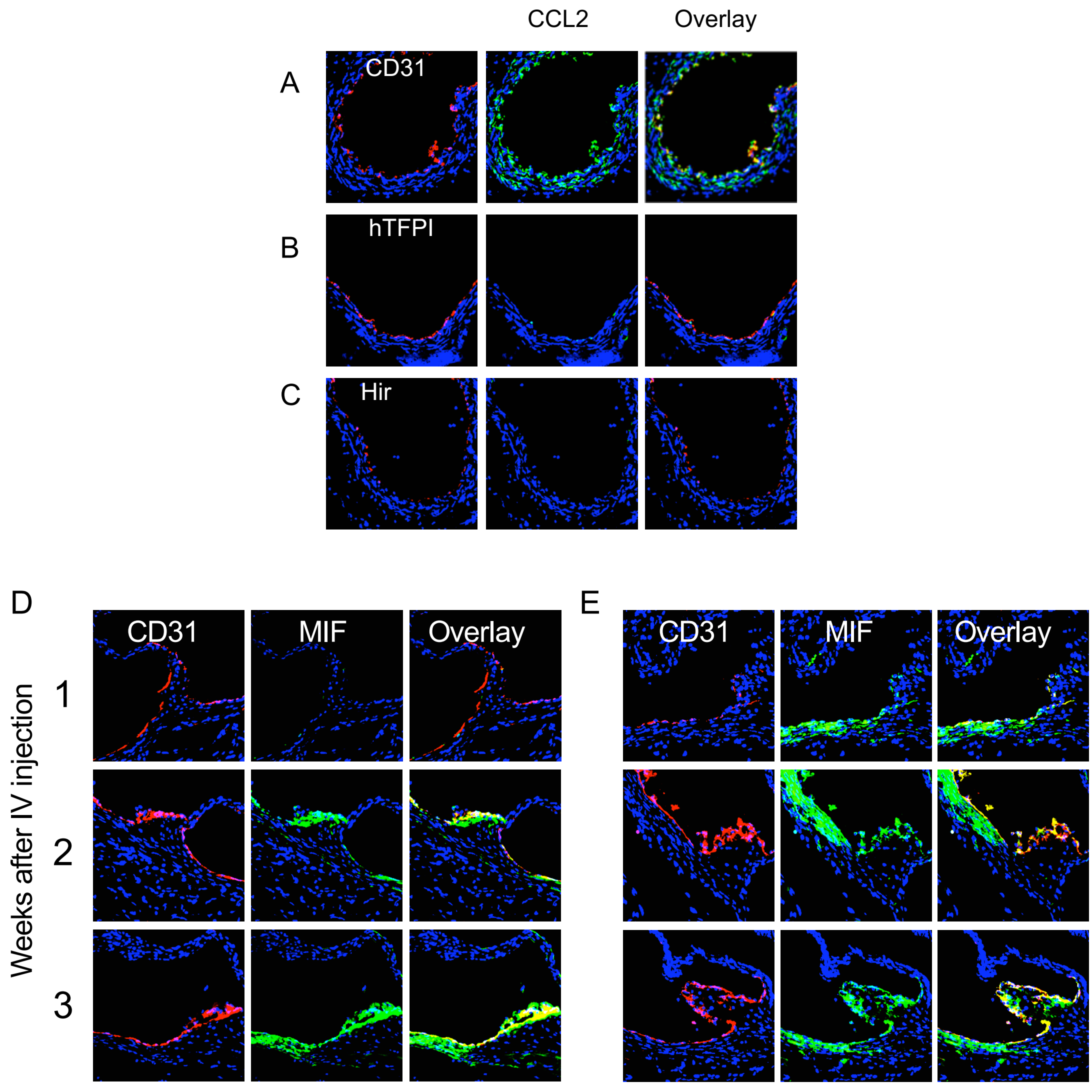

A-C. Three colour immunofluorescence images of sections through donor aortas, 6 weeks post-transplantation. Recipients were ApoE-/- mice, fed a high fat diet (HFD) for two weeks from age 6 weeks, prior to transplantation of aorta from BL/6 (A) CD31-TFPI-Tg (B) or CD31-Hir-Tg (C). Blue - nuclear stain 4',6-diamidino-2-phenylindole (DAPI). Red - anti-CD31 (A) anti-hTFPI (B) or anti-hirudin (Hir-C). Green – CCL2. Each panel of three images shows consecutive sections.

D&E: Three colour IF images of consecutive sections through aortic root, taken 1, 2 or 3 weeks post IV injection of 10μg/g of PTL060 (D) or PBS (E). ApoE-/- mice were commenced on a high fat diet 2 weeks prior to the injections. Blue - DAPI. Red - anti-CD31. Green - MIF.

Figure S3. PTL060 inhibits thrombin- and PAR-1-mediated chemokine production in vitro.

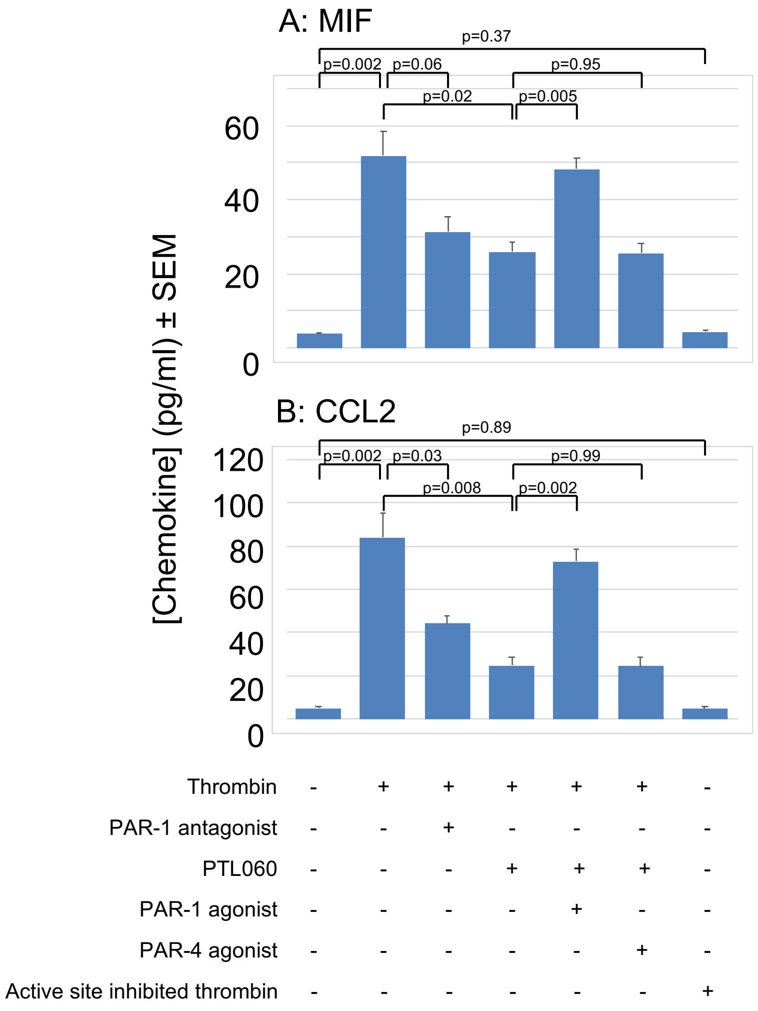

In vitro analysis of MIF (A) or CCL2 (B) production by cultured mouse SMCs, following stimulation by thrombin, with addition of reagents to demonstrate that PTL060 predominantly inhibits PAR-1 mediated chemokine production. N=3 measures per sample. Comparisons of significance by unpaired 2-tailed students t test. P<0.05 is considered significant.
Experiment repeated twice.

Figure S4. Systemic inhibition of inflammation by PTL060.

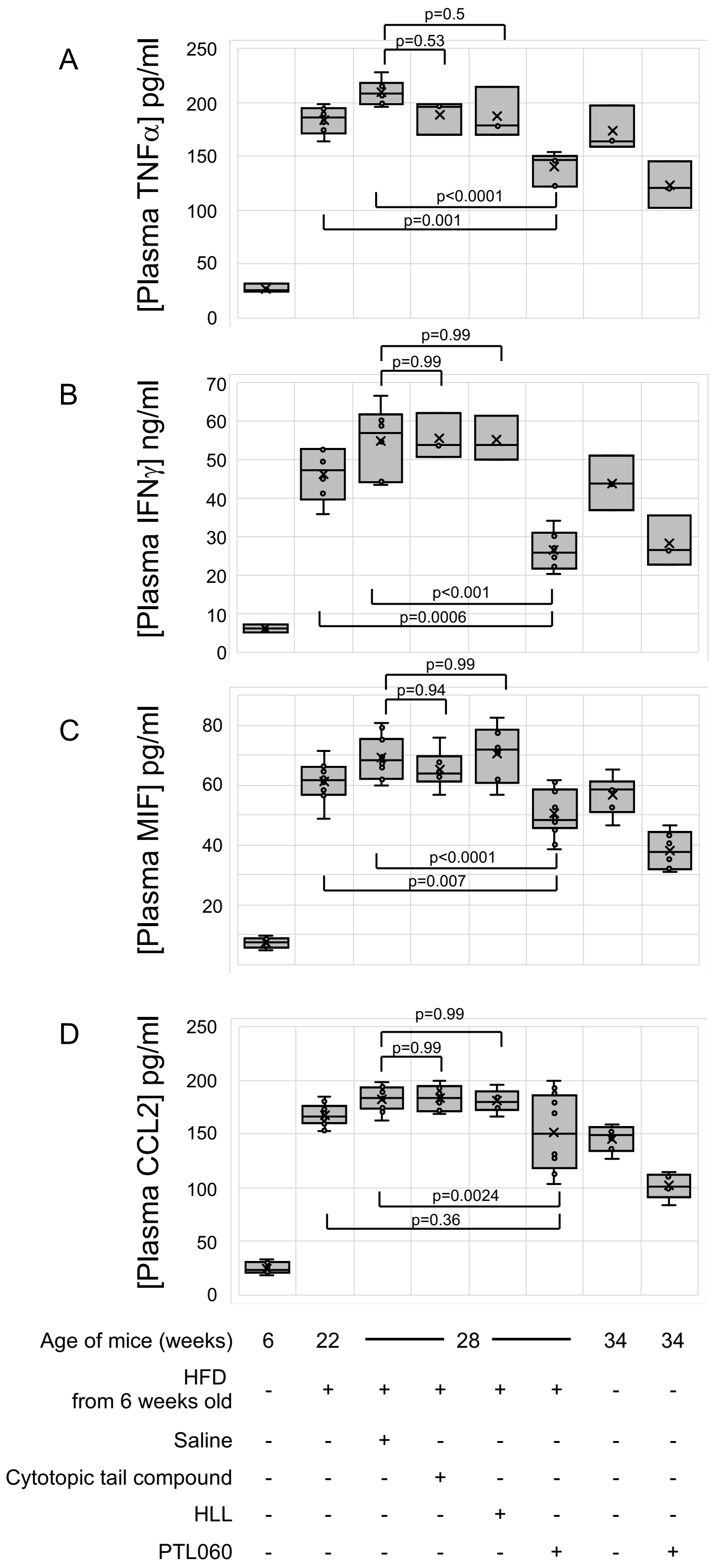

Plasma TNFα (A), IFNγ (B), MIF (C) and CCL2 (D) in different groups of ApoE-/- mice, as indicated on abscissa.

Graphs show box plots with median with interquartile range (IQR) with whiskers showing upper and lower limits and outliers indicated as single data points. Means are represented with ‘x’. Comparisons analysed by repeated measures two way Anova. Because multiple comparisons were made from these animals, $p<0.0026$ is statistically significant.

Figure S5. Phenotype of plaque cells induced by PTL060_2.

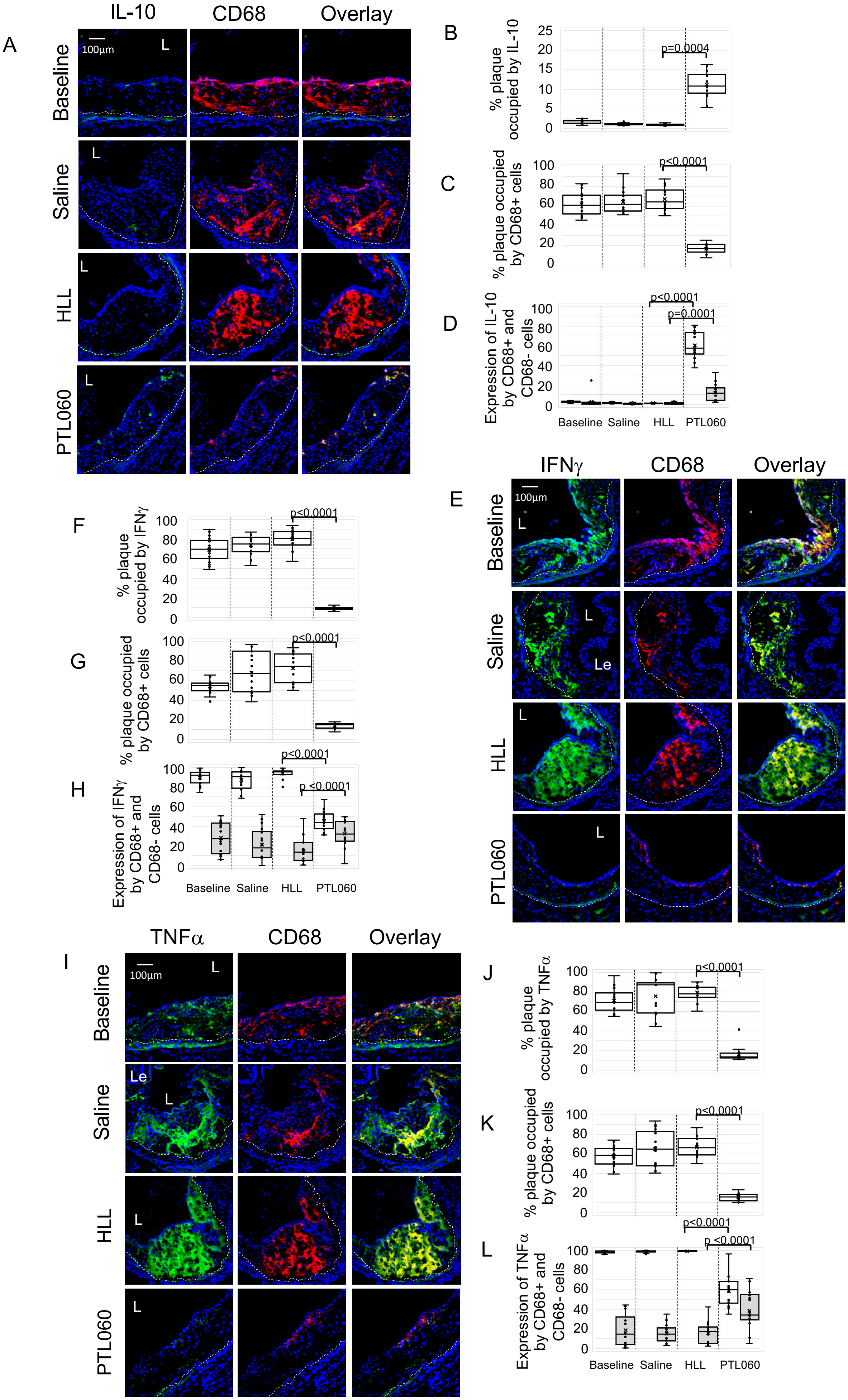

Confocal microscopic analysis of three colour immunofluorescence images through consecutive sections of aortic roots of ApoE-/- mice, fed a high fat diet from 6 to 22 weeks ('Baseline', all panels) or 6-28 weeks, with mice administered weekly injections of saline, HLL, or PTL060 as indicated between weeks 22-28. Panels show the plaque expression of CD68 (red) with (green) either IL-10 (A) IFNγ (E) or TNFα (I). Yellow in overlay image indicates co-localisation. The plaque area is demarcated by the lumen (L) and the dotted white line. Le= aortic leaflet.
Each panel of images is accompanied by graphical representations of the % of plaque area staining for the molecule of interest (B-IL-10, F-IFNγ, J-TNF α) and the % of plaque area occupied by CD68+ (C, G, K) and the proportion of CD68+ cells (white bars) and CD68-negative cells (grey bars) co-staining for IL-10 (D), IFNγ (H), or TNF α (L). Each graph is a box plot with median with interquartile range (IQR) with whiskers showing upper and lower limits and outliers indicated as single data points. Means are represented with 'x'. Each is derived from an assessment of each of the three aortic root plaques from at least 6 individual mice. Comparisons analysed by repeated measures two way Anova. Because multiple comparisons were made from these animals, $p<0.0026$ is statistically significant.

Figure S6. Phenotype of plaque cells induced by PTL060_3.

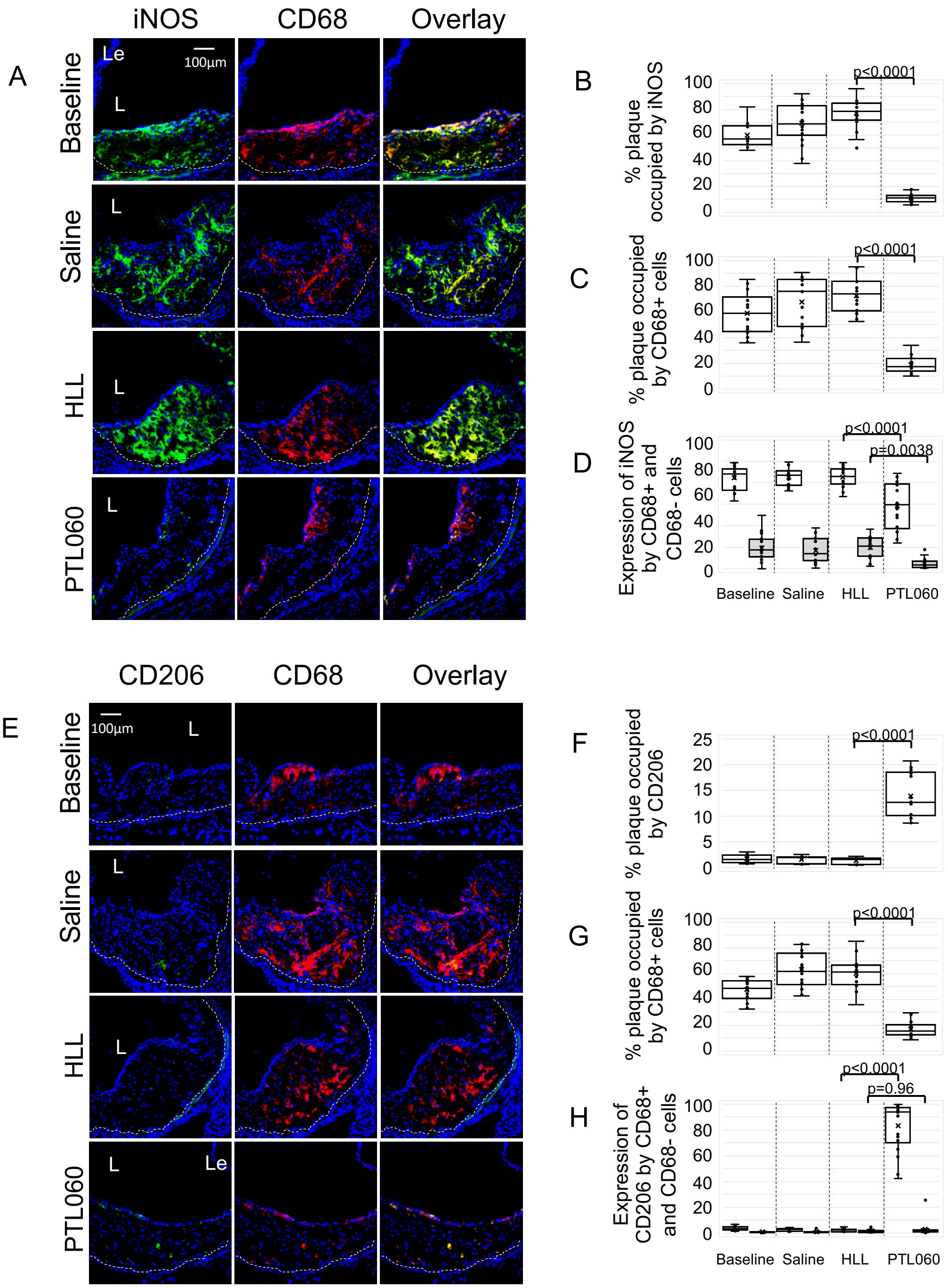

Confocal microscopic analysis of three colour immunofluorescence images through consecutive sections of aortic roots of ApoE-/- mice, fed a high fat diet from 6 to 22 weeks ('Baseline', all panels) or 6-28 weeks, with mice administered weekly injections of saline, HLL, or PTL060 as indicated between weeks 22-28. Panels show the plaque expression of CD68 (red) with (green) either iNOs (A) or CD206 (E). Yellow in overlay image indicates co-localisation. The plaque area is demarcated by the lumen (L) and the dotted white line. Le= aortic leaflet.

Each panel of images is accompanied by graphical representations of the % of plaque area staining for the molecule of interest (B-iNOS, F-CD206) and the % of plaque area occupied by CD68+ (C, G) and the proportion of CD68+ cells (white bars) and CD68-negative cells (grey bars) co-staining for iNOS (D) or CD206 (H). Each graph is a box plot with median with interquartile range (IQR) with whiskers showing upper and lower limits and outliers indicated as single data points. Means are represented with 'x'. Each is derived from an assessment of each of the three aortic root plaques from at least 6 individual mice. Comparisons analysed by repeated measures two way Anova. Because multiple comparisons were made from these animals, $p<0.0026$ is statistically significant.

Figure S7. Impact of adoptive transfer of CD11b+ cells expressing hirudin_2.

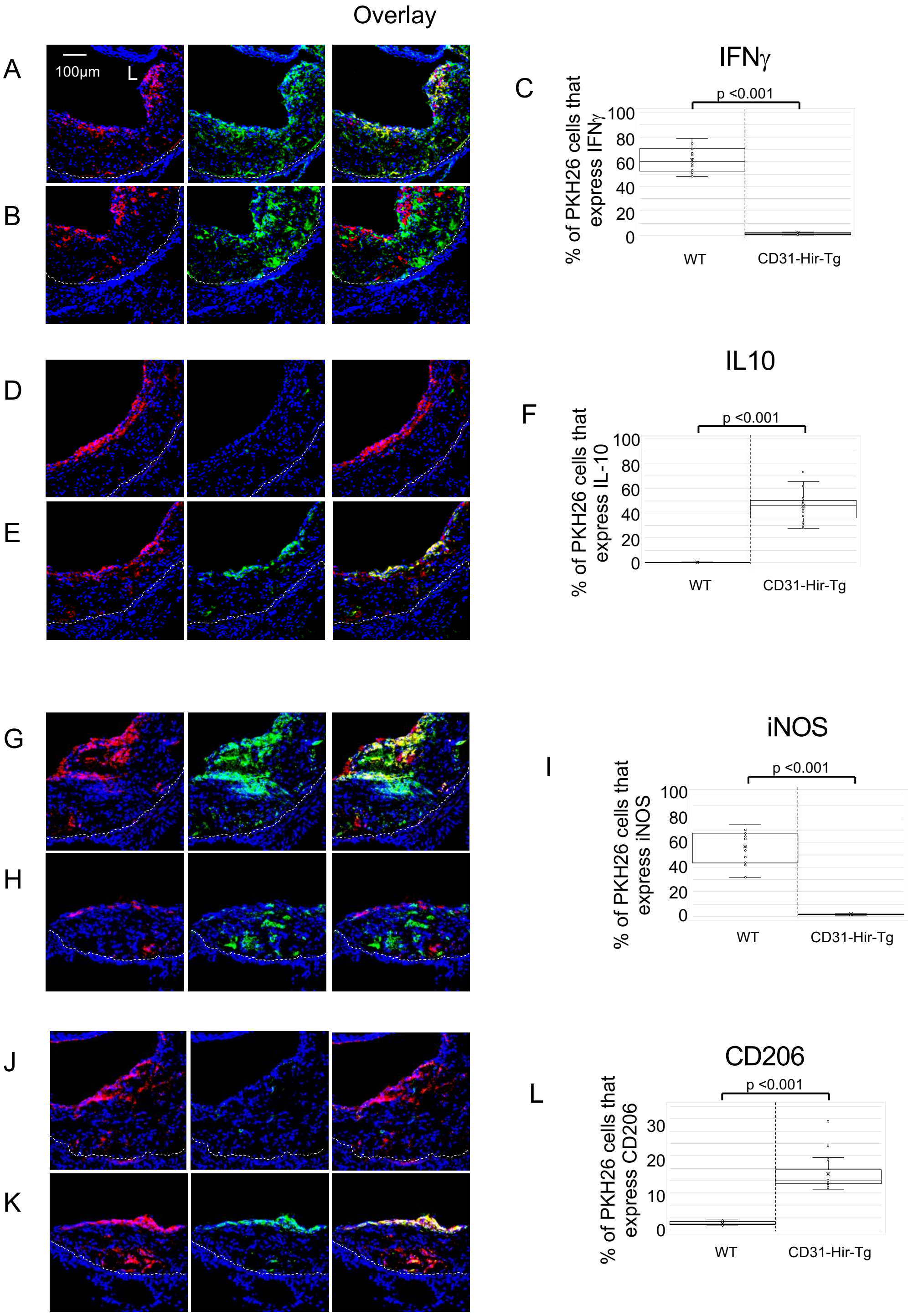

All panels: CD11b cells, harvested from either BL/6 or CD31-Hir-Tg mice were labelled in vitro with PKH26 (red) and adoptively transferred into ApoE-/- mice fed a HFD between ages of 6-22 weeks. Aortic roots were collected 48 hours post-injection, for confocal IF analysis of the phenotype of adoptively transferred cells. Graphs are a box plot with median with interquartile range (IQR) with whiskers showing upper and lower limits and outliers indicated as single data points. Means are represented with 'x'. Each is derived from a double assessment of each of the six aortic root plaques from 6 individual mice.
A-C: To illustrate expression of IFNγ (green) within the plaque after adoptive transfer of CD11b+ cells from BL/6 (A) or CD31-HIr-Tg (B) mice. (C) illustrates quantitative assessment of the proportion of PKH26+ cells co-expressing IFNγ .
D-F: To illustrate expression of IL-10 (green) within the plaque after adoptive transfer of CD11b+ cells from BL/6 (D) or CD31-HIr-Tg (E) mice. (F) illustrates quantitative assessment of the proportion of PKH26+ cells co-expressing IL-10.
G-I: To illustrate expression of iNOS (green) within the plaque after adoptive transfer of CD11b+ cells from BL/6 (G) or CD31-HIr-Tg (H) mice. (I) illustrates quantitative assessment of the proportion of PKH26+ cells co-expressing iNOS.
J-L: To illustrate expression of CD206 (green) within the plaque after adoptive transfer of CD11b+ cells from BL/6 (J) or CD31-HIr-Tg (K) mice. (L) illustrates quantitative assessment of the proportion of PKH26+ cells co-expressing CD206.
Quantitative comparisons analysed by repeated measures two way Anova. Because multiple comparisons were made from these animals, $p<0.0055$ is statistically significant.

Figure S8. Monocyte recruitment and phenotype after systemic PTL060_2.

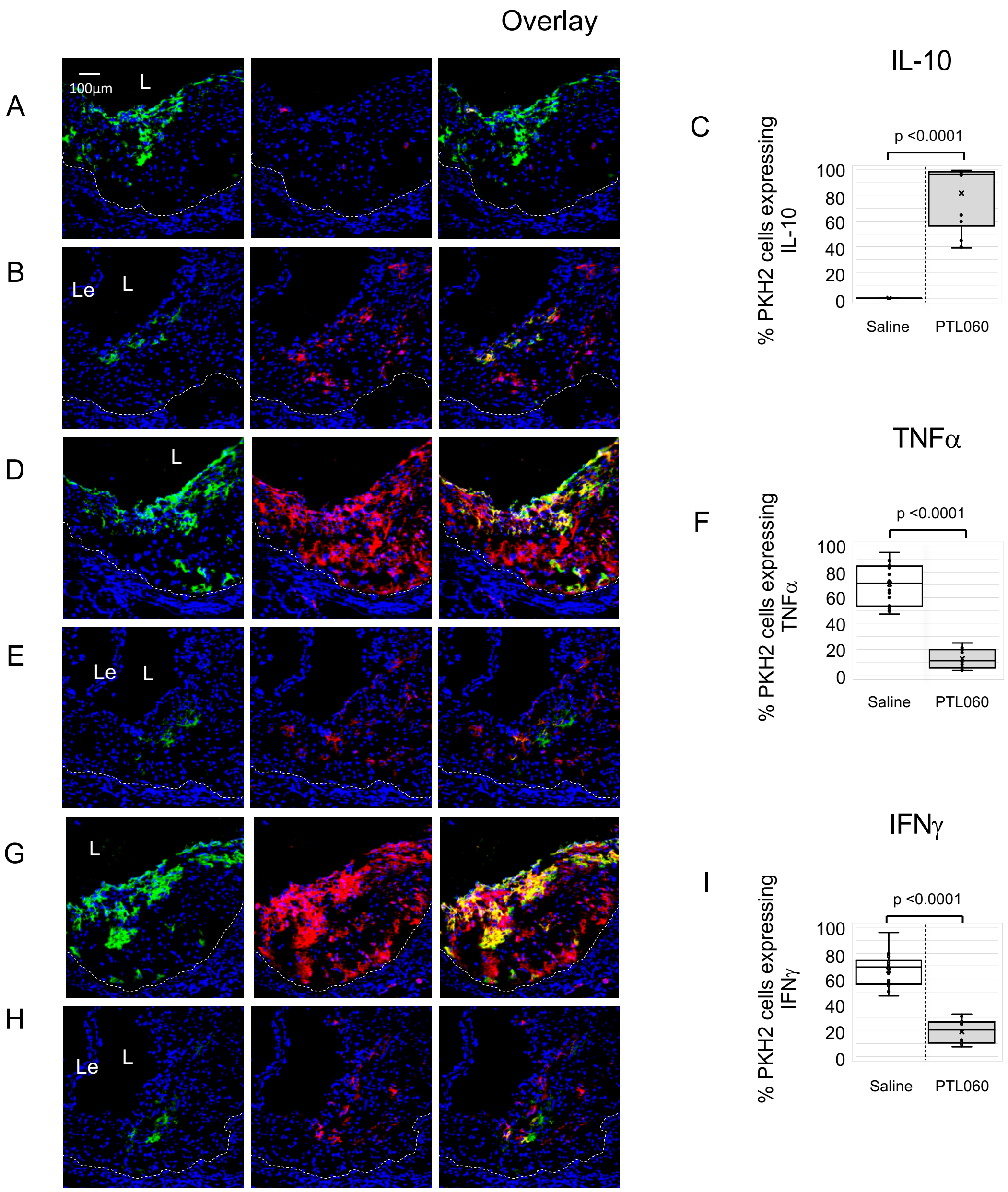

Confocal microscopic analysis of three colour immunofluorescence images through consecutive sections of aortic roots of ApoE-/- mice, fed a high fat diet from 6 to 26 weeks, with mice administered weekly injections of saline or PTL060 as indicated below between weeks 22-25. 1 week after the last injection, mice were injected with PKH2-labelled CD11b cells (green) and aortic roots harvested 48 hours later. Graphs are a box plot with median with interquartile range (IQR) with whiskers showing upper and lower limits and outliers indicated as single data points. Means are represented with 'x'. Each is derived from a double assessment of each of the three aortic root plaques from 3 individual mice.

A-C: To illustrate the expression of IL-10 (red) after adoptive transfer of BL/6 CD11b+ cells in mice treated with saline (A) or PTL060 (B). (C) illustrates quantitative assessment of the proportion of PKH2+ cells co-expressing IL-10.

D-F: To illustrate the expression of TNFα (red) after adoptive transfer of BL/6 CD11b+ cells in mice treated with saline (D) or PTL060 (E). (F) illustrates quantitative assessment of the proportion of PKH2+ cells co-expressing TNFα.

G-I: To illustrate the expression of IFNγ (red) after adoptive transfer of BL/6 CD11b+ cells in mice treated with saline (G) or PTL060 (H). (I) illustrates quantitative assessment of the proportion of PKH2+ cells co-expressing IFNγ.

Quantitative comparisons analysed by repeated measures two way Anova. Because multiple comparisons were made from these animals, $p<0.007$ is statistically significant.
